# Genetic characterization of Greek population isolates reveals strong genetic drift at missense and trait-associated variants

**DOI:** 10.1038/ncomms6345

**Published:** 2014-11-06

**Authors:** Kalliope Panoutsopoulou, Konstantinos Hatzikotoulas, Dionysia Kiara Xifara, Vincenza Colonna, Aliki-Eleni Farmaki, Graham R. S. Ritchie, Lorraine Southam, Arthur Gilly, Ioanna Tachmazidou, Segun Fatumo, Angela Matchan, Nigel W. Rayner, Ioanna Ntalla, Massimo Mezzavilla, Yuan Chen, Chrysoula Kiagiadaki, Eleni Zengini, Vasiliki Mamakou, Antonis Athanasiadis, Margarita Giannakopoulou, Vassiliki-Eirini Kariakli, Rebecca N. Nsubuga, Alex Karabarinde, Manjinder Sandhu, Gil McVean, Chris Tyler-Smith, Emmanouil Tsafantakis, Maria Karaleftheri, Yali Xue, George Dedoussis, Eleftheria Zeggini

**Affiliations:** 1 Department of Human Genetics, Wellcome Trust Sanger Institute, Hinxton CB10 1HH, UK; 2 Wellcome Trust Centre for Human Genetics, University of Oxford, Oxford OX3 7BN, UK; 3 Department of Statistics, University of Oxford, Oxford OX1 3TG, UK; 4 Institute of Genetics and Biophysics ‘A. Buzzati-Traverso’, National Research Council (CNR), Naples 80131, Italy; 5 Department of Nutrition and Dietetics, Harokopio University of Athens, Athens 17671, Greece; 6 European Molecular Biology Laboratory, European Bioinformatics Institute, Hinxton, Cambridge CB10 1SD, UK; 7 H3Africa Bioinformatics Network (H3ABioNet) Node, National Biotechnology Development Agency (NABDA), Federal Ministry of Science and Technology (FMST), Abuja 900107, Nigeria; 8 International Health Research Group, Department of Public Health and Primary Care, University of Cambridge, Cambridge CB1 8NR, UK; 9 Oxford Centre for Diabetes, Endocrinology and Metabolism, University of Oxford, Churchill Hospital, Oxford OX3 7LJ, UK; 10 Department of Health Sciences, University of Leicester, Leicester LE1 7RH, UK; 11 Division of Medical Genetics, Department of Reproductive Sciences and Development, IRCCS-Burlo Garofolo, University of Trieste, Trieste 34137, Italy; 12 Anogia Medical Centre, Anogia 74051, Greece; 13 Dromokaiteio Psychiatric Hospital of Athens, Chaidari, Athens 12461, Greece; 14 Department of Human Metabolism, University of Sheffield, Sheffield S10 2TN, UK; 15 School of Medicine, National and Kapodistrian University of Athens, Goudi, Athens 11527, Greece; 16 Echinos Medical Centre, Xanthi 67300, Greece; 17 School of Health Sciences, Faculty of Nursing, National and Kapodistrian University of Athens, Goudi, Athens 11527, Greece; 18 Medical Research Council/Uganda Virus Research Institute, Uganda Research Unit on AIDS, PO Box 49, Entebbe, Uganda

## Abstract

Isolated populations are emerging as a powerful study design in the search for low-frequency and rare variant associations with complex phenotypes. Here we genotype 2,296 samples from two isolated Greek populations, the Pomak villages (HELIC-Pomak) in the North of Greece and the Mylopotamos villages (HELIC-MANOLIS) in Crete. We compare their genomic characteristics to the general Greek population and establish them as genetic isolates. In the MANOLIS cohort, we observe an enrichment of missense variants among the variants that have drifted up in frequency by more than fivefold. In the Pomak cohort, we find novel associations at variants on chr11p15.4 showing large allele frequency increases (from 0.2% in the general Greek population to 4.6% in the isolate) with haematological traits, for example, with mean corpuscular volume (rs7116019, *P*=2.3 × 10^−26^). We replicate this association in a second set of Pomak samples (combined *P*=2.0 × 10^−36^). We demonstrate significant power gains in detecting medical trait associations.

## Abstract (web summary)

Isolated populations can increase power to detect low frequency and rare risk variants associated with complex phenotypes. Here, the authors identify variants associated with haematological traits in two isolated Greek populations that would be difficult to detect in the general population, due to their low frequency.

## Graphical abstract

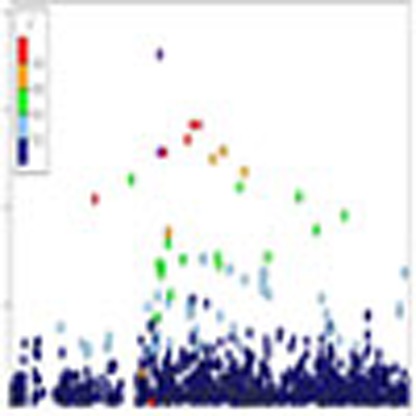

The study of isolated populations can help to increase the power of genetic association studies in search of low-frequency and rare variant associations with complex traits^1^,^2^,^3^,^4^,^5^,^6^,^7^. Population isolates have key features that can be used in disease mapping such as reduced environmental, phenotypic and genetic heterogeneity^8^. Isolated populations are usually contained within a region because of geographical and/or cultural barriers that often coincide with a homogeneous set of environmental exposures, for example, diet and lifestyle. The founding event and the limited or absent gene migration with neighbouring populations give rise to a small effective population size (Ne), which is maintained over time and results in increased levels of homozygosity and linkage disequilibrium (LD). The genetic architecture of isolates can be further influenced by population bottlenecks that decrease population size. Under these circumstances, genetic drift can lead to significant reduction of extant genetic variability and drift potentially trait-associated alleles to higher frequency.

Under the auspices of the HELlenic Isolated Cohorts study (HELIC), we have collected samples from two isolated populations in Greece: the Pomak villages (HELIC-Pomak), a set of religiously isolated mountainous villages in the North of Greece, and the Mylopotamos mountainous villages (HELIC-MANOLIS) on the island of Crete. All samples have information on anthropometric, cardiometabolic, biochemical, haematological and diet-related traits and both cohorts form the substrate for medically relevant trait association studies.

The HELIC-MANOLIS (Minoan isolates) collection comprises individuals from the mountainous Mylopotamos villages, including Anogia, Zoniana, Livadia and Gonies (estimated population size of 6,000 in total). Residents of the Mylopotamos villages have over the centuries preserved their customs, traditions and anthropological character. The Pomaks inhabit the regional units of Xanthi, Rhodope and Evros. The HELIC-Pomak collection comprises individuals from the Pomak villages located in the regional unit of Xanthi (estimated to be 25,000 in total). The Pomaks represent a small minority of Muslims in Greece with uncertain historical origins^9^. The two most dominant theories state that the Pomaks are either descendants of natives of the Rhodope mountains (on the border of Bulgaria and Greece^10^ or have come from Asia^11^). The Pomak language is thought to be either a Bulgarian or a Slavic dialect; however, today most Pomaks are also fluent in Turkish and Greek. After World War II and through the end of 1995, most Pomaks lived in a military-restricted zone, access to which required special permission^12^.

The aims of our study are twofold. First, we use genome-wide genotype data to genetically characterize the MANOLIS and Pomak cohorts and compare their allelic architecture to the general Greek population; we demonstrate that isolation caused extensive genetic drift in both isolates. Second, we evaluate the gains in power for complex trait mapping studies that can be afforded by studying well-characterized population isolates in two ways: we calculate power to detect loci that have drifted up in frequency in the isolates compared with the general Greek population highlighting significant theoretical gains in power for variants at the lower end of the minor allele frequency (MAF) spectrum. We then demonstrate this empirically by examining the biological role of these variants and their association with medically important complex traits. We find an enrichment of missense variants among alleles that have drifted up in frequency in the MANOLIS cohort and novel associations between haematological traits and alleles that have drifted up in frequency in the Pomak cohort.

## Results

### Population genetics

**Figure 1:**
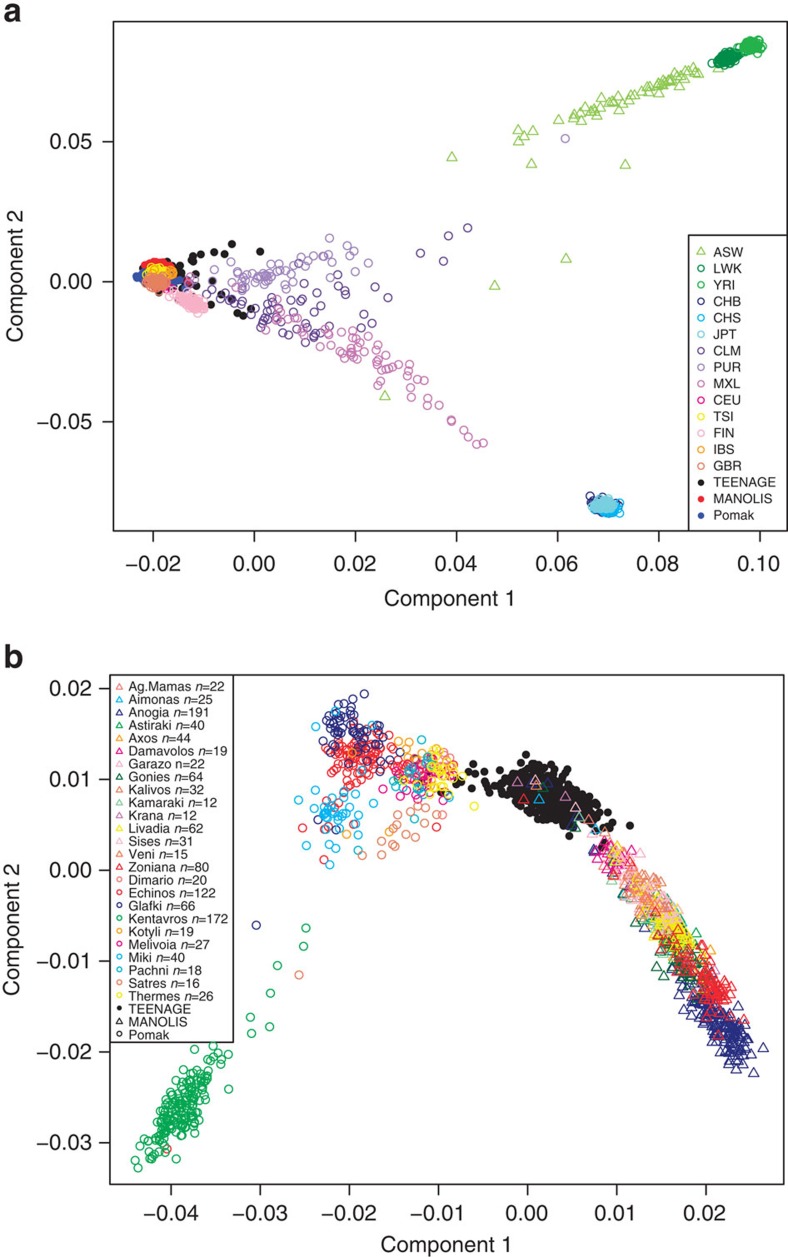
Population clustering. (**a**) MDS analysis of HELIC-MANOLIS, HELIC-Pomak and TEENAGE combined with populations from the 1000 Genomes Project. (**b**) MDS analysis of HELIC-MANOLIS villages, HELIC-Pomak villages and TEENAGE. Individuals from the MANOLIS cohort are depicted by the differently coloured hollow triangles with each colour corresponding to the village of origin. Individuals from the Pomak villages are depicted by the differently coloured hollow circles with each colour corresponding to the village of origin. The black solid circles depict individuals from TEENAGE representing the general Greek population.

To characterize the isolates in the context of broader global diversity, we applied multidimensional scaling (MDS) analyses including data from the general Greek population and from the 1000 Genomes Project^13^. As expected, the two isolates formed well-defined clusters proximal to the general Greek population (TEENAGE), the Utah Residents with Northern and Western European Ancestry (CEU) and the Toscani in Italia (TSI) population clusters (Fig. 1a). We also observed a distinctive pattern of the two population isolates clustering on either side of the general Greek population (Fig. 1b). This is consistent with the results of population differentiation assessed via pairwise Fst calculations that support higher, albeit still small, genetic differentiation between the isolates (Fst for MANOLIS versus Pomak=0.005) than between each isolate and the general population (Fst for MANOLIS versus TEENAGE=0.002 and Fst for Pomak versus TEENAGE=0.003; Supplementary Table 1). MDS analysis at this resolution reveals some intrapopulation structure for the Pomak population where individuals from one particular village (Kentavros) tend to form a distinct cluster (Fig. 1b). Since Kentavros is the largest village population in the Pomak cohort and population structure analyses can be strongly influenced by uneven sampling^14^, we repeated the MDS analysis by randomly selecting *N*=61 individuals from Kentavros, which is comparable to the size of other large villages; we observe that the subsampled Kentavros individuals do not form a distinct cluster (Supplementary Fig. 1).

### Levels of isolatedness

The inbreeding coefficient (Fin) was higher in the Pomak and MANOLIS populations compared with TEENAGE (0.014, 0.007 and 0.0004, respectively; Supplementary Table 2). Similarly, genome-wide homozygosity (Fhom) was elevated in the Pomak and MANOLIS populations compared with TEENAGE (0.643±0.006, 0.639±0.004 and 0.635±0.002, respectively), with the Pomak population showing higher variance in inbreeding than MANOLIS and TEENAGE, in which a tighter distribution is observed (Supplementary Fig. 2).

Consistent with these observations, genomic runs of homozygosity (ROH) show that the two isolated populations have on average a much larger number (nROH) and length (cROH) of ROH than TEENAGE. The Pomak isolate displays higher ROH values with respect to the MANOLIS isolate (Supplementary Table 2; Supplementary Fig. 3). When using a different definition of calling ROH based on LD-pruned data, we observe a larger relative difference in nROH and cROH in the two isolates compared with TEENAGE (Supplementary Table 2). This is expected, given that the ROH detected using the latter approach are likely to be because of recent parental relatedness rather than ancient patterns of shared ancestry.

**Figure 2:**
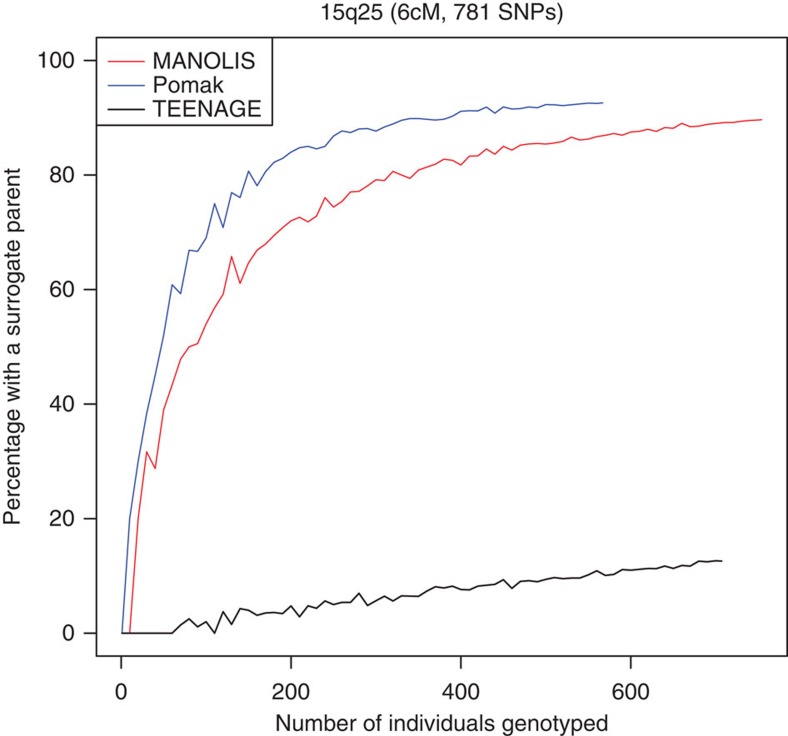
Proportion of individuals with at least one ‘surrogate parent’ in each study population. Surrogate parents refer to individuals from the same population who share local haplotypes with the index individual. The proportion of surrogate parents is shown for each of the two population isolates, MANOLIS and Pomak, and the general Greek population, TEENAGE, as a function of sample size for a region of the genome (chr15q25).

We computed for each population the proportion of individuals that have at least one ‘surrogate parent’ for randomly chosen regions in the genome of varying length^15^. This concept was coined by Kong *et al*.^15^ and refers to individuals from the same population who share local haplotypes with the index individual; these can be used to phase the index individual genotypes as though they are actual parents. Our results for randomly chosen regions across the genome are qualitatively similar. For example, for a region on 15q25 (781 single-nucleotide polymorphisms (SNPs)) we observe that with as little as 300 individuals analysed per population 88% of Pomak individuals and 79% of MANOLIS individuals have at least one ‘surrogate parent’ compared with 6% in the general Greek population (Fig. 2).

**Figure 3:**
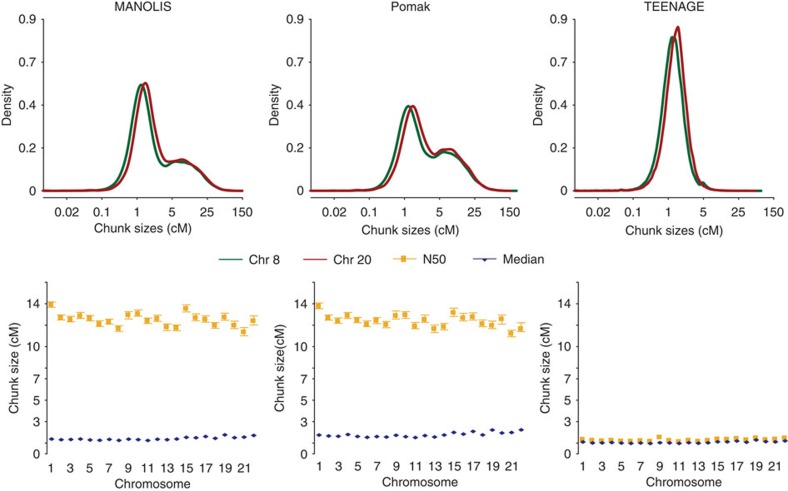
Maximal haplotype sharing between nearest-neighbours (NNs) for each cohort. The output of our algorithm is a set of shared haplotypes of different lengths. The top panel shows the density curves for the shared haplotype chunks for chromosomes 8 (dark green) and 20 (dark red). Results were similar for the other chromosomes. The bottom panel shows the per chromosome median (dark blue) and N50 (gold), in cM, of all shared haplotype chunks obtained from all the unrelated individuals (pi hat<0.2, see Methods) in each cohort. The 95% confidence intervals (CIs), obtained by bootstrapping using 1000 repeats of resampling without replacement, are denoted by the horizontal bars (where these are very tight they are not visible in the plot). The median chunk length tells us about the distribution of contigs; however, we may also be interested in what a randomly sampled location will look like. The N50 measures this; specifically, it is a chunk length such that 50% of all randomly chosen positions will fall in a chunk of at least that size.

Using the modified long-range phasing (LRP) method^15^ (Supplementary Methods) we find a much greater extent of haplotype sharing in the isolated populations, with the median length of haplotypes being 1.78, 1.42 and 1.07 cM in the Pomak, MANOLIS and TEENAGE cohorts, respectively (Fig. 3). Consequently, 50% of randomly sampled locations within the genome lie in shared haplotype lengths of at least 12.4, 12.5 and 1.3 cM, respectively (N50 haplotype length; Fig. 3). Interestingly, the distribution of shared haplotype lengths in the isolates is bimodal (Fig. 3), with peaks corresponding to very recent co-ancestry (within the last few generations) and much older co-ancestry (as in the TEENAGE population). Since only unrelated samples have been used in this analysis, it is likely that this difference reflects co-ancestry dating prior and subsequent to the population founding event, respectively.

The extent of isolation of the Pomak and MANOLIS samples is further reflected in the fact that nearest neighbours (NNs), that is, genealogically closest individuals in the sample (Supplementary Methods), are typically chosen from within their cohort: 88.3% and 90.5%, respectively, versus 48.5% for the TEENAGE samples (Supplementary Fig. 4). While ~10% of the isolates have NNs from the TEENAGE cohort, the shared haplotype chunks specific to these NNs are significantly shorter, fourfold and threefold for the Pomak and MANOLIS cohorts, respectively (Supplementary Fig. 5), suggesting that typically the inferred co-ancestry pre-dates the isolation events. The limited number of larger haplotype chunks (0.5% of chunks in the Pomak cohort copied from TEENAGE are >5 cM, 1% for MANOLIS; Supplementary Fig. 5) is indicative of very limited migration and gene flow events.

**Figure 4:**
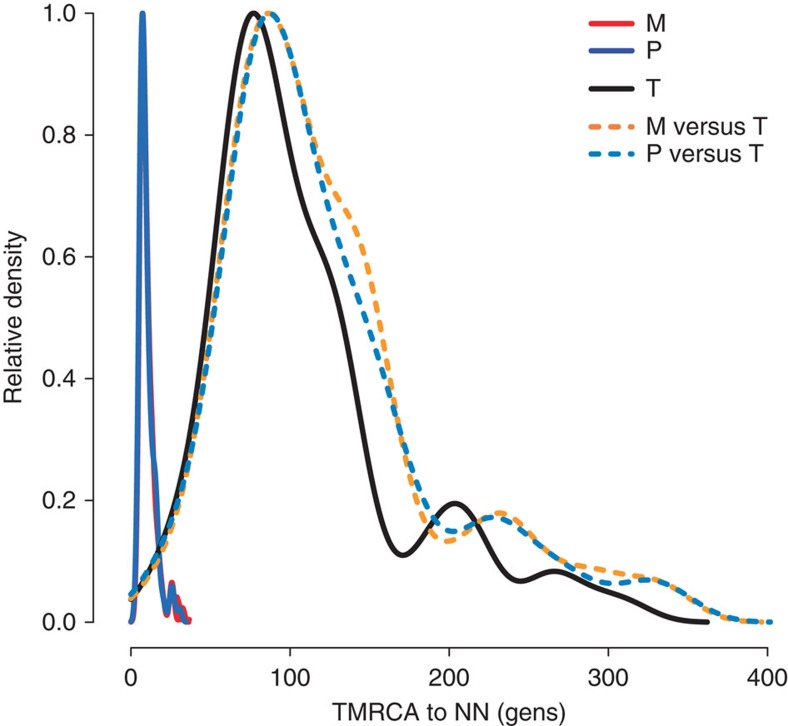
Relative density curves showing the estimated number of generations to the common ancestor between an individual and their NN. The results are from 100 randomly sampled SNPs from across the genome for the MANOLIS individuals (M), the Pomaks (P), the TEENAGE samples (T), each considered separately and a scenario where the MANOLIS individuals (M versus T) and the Pomaks (P versus T) each are forced to select NNs from the TEENAGE cohort.

To estimate the age of co-ancestry, we fit a model of exponential decay of haplotype sharing around 100 randomly sampled locations across the genome (Supplementary Fig. 6; Supplementary Methods). We find that the median age of common ancestors is 8.56 and 8.65 generations in Pomak and MANOLIS, respectively, but 89.7 generations in TEENAGE (Fig. 4), which is comparable to that observed in other European populations. Using a simple moment estimator, these values imply recent effective population sizes of 2,400, 3,200 and 32,000 for the three populations, respectively. In short, the rate of genetic drift in the isolated populations is ~10 times that in the TEENAGE population.

Using a different approach that utilizes pairwise LD information^16^,^17^,^18^, we observe a qualitatively similar result (Supplementary Fig. 7, Supplementary Note 1). By examining trends of effective population size through time, we observe a recent expansion in TEENAGE as observed in general European populations^19^,^20^, whereas the two isolates show a reduction in effective population size, a sign that they experienced a strong genetic drift, as observed in other European isolated populations^21^.

### Genetic drift

Overall, the MANOLIS, Pomak and TEENAGE cohorts have similar allele frequency distributions (Supplementary Note 2, Supplementary Fig. 8). We calculated absolute allele frequency differences by comparing the allele frequencies of all variants in each of the two isolates against their respective allele frequency in the general Greek population. Compared with the general Greek population, we find that 49.74% (324,689) and 48.59% (311,922) of variants examined genome wide have increased in frequency in MANOLIS and Pomak, respectively (Supplementary Table 3). In all, 50.03% (326,623) and 51.19% (328,629) of variants examined genome wide have decreased in frequency in MANOLIS and Pomak, respectively, and 0.23% of variants in each isolate showed no allele frequency change with respect to TEENAGE (Supplementary Table 3).

In the respective fold analyses, 517 (0.08%) variants displayed >10-fold increase in MANOLIS versus TEENAGE (Table 1). The highest fold difference in MANOLIS versus TEENAGE was observed for rs4135336, an intronic variant in *PPARG* (peroxisome proliferator-activated receptor gamma). Rs4135336 shows an increase in allele A frequency by 47-fold (from 0.0007 in TEENAGE to 0.033 in MANOLIS). This variant appears to be monomorphic in all European (EUR) populations from the 1000 Genomes Project except in TSI where it has been observed at a frequency of 0.01. It is, however, common in African (AFR) populations and has reached 0.14 frequency in the Yoruban (YRI) population.

A total of 261 (0.04%) variants displayed an increase of >10-fold in Pomak versus TEENAGE and two variants displayed an increase of >50-fold (Table 1). The highest fold difference was observed for rs12173144, an intergenic variant located between *CDH9* (cadherin 9, type 2 (T1-cadherin)) and *CDH6* (cadherin 6, type 2, K-cadherin (fetal kidney)) that shows an increase in allele G frequency by 59-fold (from 0.0007 in TEENAGE to 0.0423 in Pomak). This variant is observed at low frequency (0.01–0.03) in all EUR populations from the 1000 Genomes Project but is common in populations from East Asia (ASN), Ad Mixed America (AMR) and AFR observed at the highest frequency in YRI (0.22). Rs9958680, an intronic variant in *SLC14A2* (solute carrier family 14 (urea transporter), member 2), shows an increase in allele G frequency by 56-fold (from 0.0007 in TEENAGE to 0.03897 in Pomak). This variant is monomorphic in all EUR and ASN populations from the 1000 Genomes Project, has low frequency (0.01–0.02) in AMR populations and is common in populations from AFR, observed at the highest frequency in the Luhya (LWK) population (0.18).

### Power calculations

To investigate power gains for frequency differences at rare alleles we examined variants that had MAF<0.01 in TEENAGE. In all, 24,493 of the variants that overlap between TEENAGE and MANOLIS are rare in TEENAGE. Of these, 15,341 (62.6%) have risen in frequency in MANOLIS and 4,581 (18%) have reached MAF>0.01. In the latter, allele frequency increases in the range from 0.000038 (MAF in MANOLIS=0.01001, MAF in TEENAGE=0.009972) to 0.06897 (MAF in MANOLIS=0.06897, MAF in TEENAGE=0) with a median increase of 0.01 (MAF in MANOLIS=0.01527, MAF in TEENAGE=0.00495). We calculated power (Supplementary Methods) to detect association in the non-isolated and isolated populations at allele frequencies that corresponded to the minimum, median and maximum values of this range and find absolute power gains of 0.4%, 76% and 100%, respectively (Supplementary Note 3). Similarly, we calculate power gains of 3.6–100% in the Pomak population (Supplementary Note 3). We note that we performed these power calculations (which assume independence of samples) by fixing the sample size to the full set of samples (including related) from our isolated populations, since our associations were carried out in the full set of samples but accounting for relatedness. We repeated the power calculations based on the sample size of unrelated individuals only in the Pomak and MANOLIS collections (Supplementary Methods) and calculate power gains of 0.1–100% in the MANOLIS and 0.5–100% in the Pomak population (Supplementary Note 3).

### Complex trait association signals

**Figure 5:**
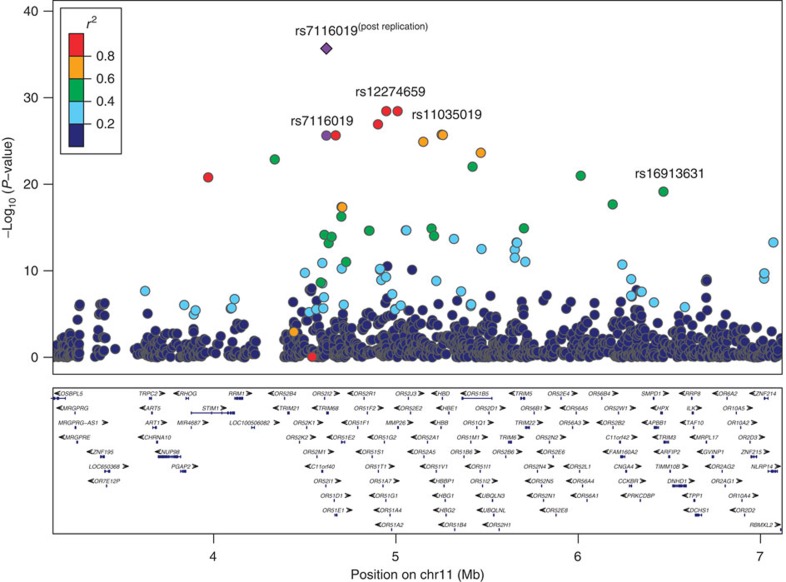
Association of variants at chr11p15.4 with MCV. The *x* axis shows the genomic interval, the left *y* axis shows the statistical significance of association as negative log10 of *P* values (score test in GEMMA). rs7116019 was used as the reference SNP and its *P* value in the discovery set and post replication is denoted by the purple circle and diamond, respectively. In addition, annotated are variants that show the highest genetic drift (rs16913631) and most significantly associated with MCV (rs12274659 and rs11035019). The linkage disequilibrium was calculated dynamically from our own data and is presented as pairwise *r*^2^ between the reference SNP and the other SNPs in the region with colours according to different bins (0–0.2, dark blue; 0.2–0.4, light blue; 0.4–0.6, green; 0.6–0.8, orange; 0.8–1.0, red). The plot was produced using LocusZoom^68^.

After ranking all variants genome wide according to their absolute or fold allele frequency differences between each isolate and the general Greek population, we investigated the tails of the distribution (top 30 variants) for associations with medically relevant traits in the full set of Pomak and MANOLIS samples using GEMMA^22^, which accounts for relatedness. In the MANOLIS cohort, none of these variants are strongly associated with any of the measured traits. In the Pomak cohort two chr11p15.4 variants (rs16913631 and rs7116019; *r*^2^=0.54) show genome-wide significant association with haematological traits. Rs16913631 allele A shows a 32-fold increase in frequency, from 0.0014 in TEENAGE to 0.0441 in Pomak. This variant appears to be monomorphic in all EUR populations from the 1000 Genomes Project but common (0.13) in AFR populations, observed at highest frequency (0.21) in LWK. Rs16913631 is associated with three haematological traits: mean corpuscular volume (MCV) in 965 Pomak individuals (for allele A: beta=−1.046, s.e.=0.106, *P*=6.86 × 10^−20^); mean corpuscular haemoglobin concentration (MCHC) in 974 individuals (beta=0.864, SE=0.106, *P*=2.75 × 10^−14^) and the mean corpuscular haemoglobin (MCH) in 960 individuals (beta=−0.606, s.e.=0.111, *P*=8.89 × 10^−8^; Supplementary Tables 4–6). Similarly, rs7116019 allele G shows a 29-fold increase in frequency, from 0.0014 in TEENAGE to 0.0415 in Pomak. This variant also appears to be monomorphic in all EUR populations from the 1000 Genomes Project but is common in AFR populations; in YRI and LWK it has a frequency of 0.18. The variant is associated with MCV (for allele G: beta=−1.239, s.e.=0.105, *P*=2.26 × 10^−26^; Fig. 5) and explains 3.1% of the trait variance, MCHC (beta=0.933 s.e.=0.107, *P*=7.06 × 10^−16^) and MCH (beta=−0.775, s.e.=0.111, *P*=1.79 × 10^−11^; Table 2 and Supplementary Tables 4–6).

The chr11p15.4 region harbours other, more significant associations with haematological traits in the Pomak cohort at rs12274659 and rs11035019 (in perfect LD with each other; *r*^2^=0.68 with rs16913631 and *r*^2^=0.86 with rs7116019; Fig. 5). The T allele of rs11035019 has increased in frequency by 22-fold (from 0.0021 in TEENAGE to 0.0459 in Pomak) and is monomorphic in the 1000 Genomes Project data except in AMR and in AFR subpopulations, in which it is observed at a frequency of 0.01–0.05. Similarly, the G allele of rs12274659 has risen in frequency by 16-fold (from 0.0028 in TEENAGE to 0.0459 in Pomak) and is only observed at a high frequency in the 1000 Genomes Project AFR populations (frequency of 0.07–0.13). Rs11035019 and rs12274659 are associated with MCV (for allele T of rs11035019 and allele G of rs12274659: beta=−1.249, s.e.=0.099 and *P*=3.45 × 10^−29^), MCHC (beta=1.010, s.e.=0.100 and *P*=5.54 × 10^−20^) and MCH (beta=−0.732, s.e.=0.106 and *P*=2.38 × 10^−11^; Supplementary Tables 4–6).

To determine whether the signal in chr11p15.4 region is because of a single or multiple independently associated variants, we tested the most highly MCV-associated index variant, rs11035019, conditioning on the two other most highly drifted variants, rs7116019 and rs16913631. Conditioning rs11035019 on rs7116019 resulted in attenuation of the signal (*P*=2.9 × 10^−05^ versus *P*=3.45 × 10^−29^, respectively) suggesting that rs7116019, rs11035019 and its perfectly correlated proxy rs12274659 are part of the same signal. Conditioning rs11035019 on rs16913631 also resulted in a higher *P* value; however, the rs11035019 association with MCV still remained strong (*P*=8.8 × 10^−13^), which that cannot conclusively rule out the presence of two signals in this region.

Previous reports have provided evidence for association of several variants with haematological and related traits at the 11p15.4 locus^23^,^24^,^25^,^26^,^27^,^28^,^29^,^30^,^31^,^32^,^33^,^34^,^35^. The association of rs12274659 and rs11035019 with MCV after conditioning on the published variants remains genome-wide significant (*P*<4.4 × 10^−18^).

### Replication

We sought replication of the chr11p15.4 signal in a second set of 734 Pomak samples genotyped on a different platform (Supplementary Methods). Among the most highly associated variants, rs7116019 was the only directly typed variant present in the Pomak replication set and was found to be significantly associated with all three traits (Table 2). After pooling the genotypes at this SNP across the two data sets and re-analysing using GEMMA^22^, which accounts for relatedness, the associations became more significant: p_combined=2.0 × 10^−36^, 8.3 × 10^−27^ and 1.7 × 10^−13^ for MCV, MCHC and MCH, respectively (Table 2). Adjustment for the first 10 principal components generated from either the discovery or replication data sets (Supplementary Methods) does not attenuate the associations (Supplementary Table 7). Further evidence that rules out a spurious association because of the subtle population structure observed among the Pomak villages is that carriers of the minor allele (*N*=92) are spread across villages (Supplementary Fig. 9).

Motivated by the common frequency of the drifted alleles in African populations, we investigated association between variants in this chr11 region (1 Mb upstream and downstream of the most highly drifted variants) and MCV, MCHC and MCH in the Ugandan General Population Cohort^36^ study of 1,479 individuals (Supplementary Methods). The four highlighted variants (MAF=0.063–0.156 in the Ugandan population) are not significantly associated with these traits in the Ugandan cohort (Supplementary Tables 4–6). The most highly associated SNP with MCV and MCH (rs73404549, *P*=8.54 × 10^−5^, not present in our data) is not in LD with the four highlighted variants (*r*^2^<0.0008) in the Ugandan cohort. Similarly, the most highly associated SNP with MCHC (rs79639430, *P*=1.10 × 10^−5^, not present in our data) is not in high LD with the four highlighted variants (*r*^2^<0.0007) in the Ugandan cohort.

### Evolutionary insights into the trait-associated variants

The haplotype length shared by all Pomak chromosomes carrying the minor alleles of the three correlated variants (rs7116019, rs12274659 and rs11035019) is ~1.8 Mb, which is unusually long given the allele frequency (Supplementary Methods, Supplementary Note 4, Supplementary Fig. 10). This suggests that positive selection could have acted on it. Since the longest segments shared between the Pomak haplotype and other populations were 183 and 230 kb with the LWK, and 7 kb with the TEENAGE, and the frequency and diversity of the haplotypes carrying the minor alleles were both highest in the LWK, this suggested a scenario where independent haplotypes had entered the two Greek populations from the LWK. We therefore modelled the entry of a variant into the Pomak population and first assessed how likely it was to reach the currently observed frequency by drift from an initial frequency of 0.01 (Supplementary Methods, Supplementary Fig. 11). This corresponds to 30 identical copies of a single allele entering the Pomak population, although it is perhaps more likely that only one copy entered. The time allowed for it to increase in frequency by drift was the maximum possible in this demographic model. Thus, the simulation is highly conservative for our purpose. Nevertheless, we find that the probability for one locus to drift from a frequency of 0.01–0.046 or over is 0.19%, and thus unlikely to occur by chance. We then performed simulations with positive selection on this allele, using selection coefficients *s*=0.005, 0.007 and 0.01 acting on carriers. Here the probabilities of reaching the observed 0.046 frequency were higher: 0.83%, 1.45% and 2.85%, respectively. We therefore conclude that positive selection is likely to have acted on the Pomak haplotype. If a 1% chance of reaching the observed frequency of 0.046 is used as a criterion, then a selective coefficient between 0.005 and 0.007 is suggested given the demographic model used here.

### Enrichment of missense variants

**Figure 6:**
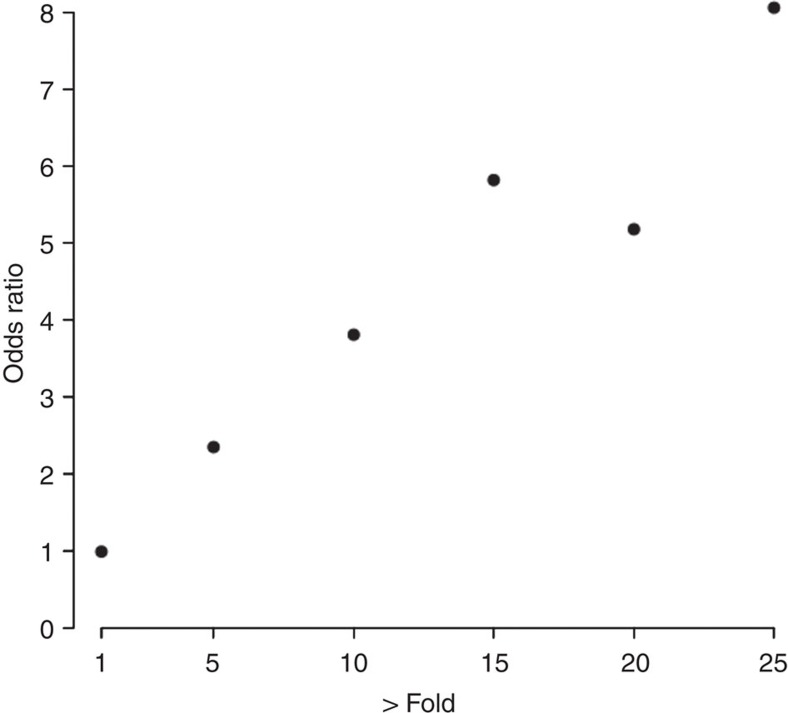
Enrichment of missense variants among those variants that have increased in frequency above incremental fold change thresholds in MANOLIS versus TEENAGE analysis. At each threshold, we computed a contingency table comparing the number of missense and other variants found at a fold change higher than the threshold. Odds ratios were used to quantify enrichment. For example, an odds ratio of 2.35 above a fold change threshold of 5 indicates that variants that have drifted in frequency by more than fivefold in the MANOLIS isolate compared with the TEENAGE general population are 2.35 times more likely to be missense than variants below this threshold.

In the MANOLIS cohort we found a significant enrichment of missense variants among those variants that have increased in frequency above a fold change of 5, and all higher thresholds examined (Fig. 6 and Supplementary Table 8). We also find a significant but considerably smaller enrichment of synonymous variants in the set of variants above fold change thresholds of 5 in MANOLIS (Supplementary Table 9). In the Pomak cohort we also observe a significant enrichment at a fold change threshold of 5; however, unlike in MANOLIS, we do not observe significant enrichment at higher thresholds or any enrichment of synonymous variants. In the MANOLIS versus TEENAGE fold analysis, there were four missense variants with a frequency increase over 25-fold: rs3741379 located in *SIPA1* (signal-induced proliferation-associated 1), rs6659553 in *POMGNT1* (protein O-linked mannose beta1,2-N-acetylglucosaminyltransferase) and two variants that are classified as deleterious in SIFT^37^: rs2274896 in *LSM14A* (SCD6 homologue A) and rs2290959 in *ACY3* (aspartoacylase (aminocyclase) 3).

### Inbreeding depression

In the Pomak population, we detected evidence for inverse associations between Fhom and height (beta=−19.755, s.e.=7.306, *P*=0.00707); Fhom and high-density lipoprotein (beta=−15.588, s.e.=6.960, *P*=0.026); Fhom and homeostatic model assessment β-cell function (HOMA_b; beta=−30.604, s.e.=15.184, *P*=0.046); Fhom and MCH (beta=−14.777, s.e.=7.051, *P*=0.037); and Fhom and MCV (beta=−17.875, s.e.=7.019, *P*=0.011; Supplementary Table 10). The associations of Fhom with these traits in MANOLIS were not significant at *P*<0.05; however, the direction of the effect was consistent with that in the Pomaks and in TEENAGE (Supplementary Table 10). We also performed this analysis using an inbreeding metric derived from ROH^38^ but find no qualitative difference in *P* values between the two analyses (Supplementary Table 10).

## Discussion

The historical origins of the Greek population isolates under study are largely unknown and the sparse historical information and genetic analyses to date have yielded inconsistent results. Modern Cretans as a whole are thought to be the descendants of Minoans who established the first advanced Bronze Age civilization in Europe about 5,000 years ago. Archaeological studies have proposed North African^39^,^40^, Cycladic^41^, Anatolian^41^,^42^ and Middle Eastern origins^43^,^44^. Genetic studies in modern Cretan populations using Y-chromosomal or mitochondrial DNA to infer ancient ancestry of the Bronze Age Cretans have indicated Anatolian^45^, Middle Eastern and Balkan^46^,^47^ origins. A recent mitochondrial DNA study refuted the hypothesis of North African Ancestry, and Minoans were found to show the strongest relationships with Neolithic Spanish, Neolithic Italian and modern European populations as well as with the modern inhabitants of the Lassithi plateau in Central Crete^48^. Our MDS results are in agreement with the MANOLIS isolate showing genetic proximity to EUR populations. The historical origins of the Greek Pomak population are even more uncertain as Pomaks are spread across Bulgaria, Turkey and Greece. Our MDS analysis using populations from the 1000 Genomes Project shows that the Pomak cohort clusters with EUR populations but cannot (nor is intended to) distinguish between these closely related ancestral populations.

All metrics used to characterize the levels of isolatedness in the HELIC cohorts (including inbreeding coefficients, ROH and the extent of haplotype sharing compared with the general Greek population) show consistently that they bear the genomic characteristics of genetic isolates. The extensive haplotype sharing in the isolates is further corroborated in the LRP analysis where for the 15q25 region we observe that with as little as 300 individuals analysed per population 88% of Pomak individuals and 79% of MANOLIS individuals have at least one ‘surrogate parent’ compared with 6% in the general Greek population. In the Icelandic population, the sample size required to reach such a high proportion of individuals with at least one surrogate parent is ~6,300 samples^15^.

In our investigation of inbreeding effects on quantitative traits, we found the most significant association between height and genome-wide homozygosity in the Pomak population isolate. This inverse association is consistent with previously reported findings in other isolated populations^49^,^50^. In keeping with our observation, McQuillan *et al*.^49^ identified effect heterogeneity across the different populations examined and argued that this could reflect biological heterogeneity. Observing an effect may depend on the variance in homozygosity in the population as well as the chance inheritance of individual recessive genotypes. It is therefore plausible that the inbreeding effect on height was detected with significance in the Pomak population because it appears more isolated but displays larger variance in homozygosity than in MANOLIS and TEENAGE. Our results support the hypothesis that along with a large number of common variants influencing height in an additive manner, multiple rare variants can also influence human height. It is noteworthy that in the Pomak population we observe evidence of inbreeding depression on MCV and MCH.

We find novel genome-wide significant association between rare variants that have drifted up in frequency in the Pomak population and MCV, MCHC and MCH at 11p15.4, which encompasses the β-globin locus. We pooled genotypes at rs7116019 across the discovery and replication data sets and repeated the association analysis appropriately accounting for sample relatedness; we find stronger evidence of association in the combined Pomak set: *P*=2.0 × 10^−36^, *P*=8.3 × 10^−27^, *P*=1.7 × 10^−13^ for the three traits, respectively. Rs7116019 is located at the 3′ end of the *TRIM68* gene that functions as an ubiquitin E3 ligase and can act as a coactivator of androgen receptor depending on its ubiquitin ligase activity^51^. Previous reports have highlighted several independent variants at the chr11p15.4 locus to be associated with anaemias, traits underlying haemoglobin composition, red blood cell indices and the clinical manifestation of thalassemias^24^,^25^,^27^,^29^,^30^,^31^,^32^,^33^. Previously a mutation in the *HBB* (haemoglobin, beta) gene, HbO-Arab (E122K), has been reported to have drifted up in frequency in the Pomak population and appears to have originated on a local haplotype about 2,000 years ago^52^. HbO-Arab leads to microcytosis and elevated MCHC^53^. The specific variant (rs33946267 located at 11:5246908) is not present in our genotype data; the variation is multiallelic and the specific allele that leads to the HbO-Arab mutation (T) is not observed in 1000 Genomes Project and NHLBI Exome Sequencing Project data; therefore, we are not able to evaluate LD with this variant.

It is noteworthy that the associated variants are monomorphic in other European populations but have risen to high frequency in African populations. The prevalence of certain haemoglobinopathies is high in the Mediterranean region and Africa^54^ and selection due to malaria is thought to be the force responsible for this^54^. We investigated association with haematological traits in a population-based cohort from Uganda but did not find evidence for association of the Pomak trait-associated variants. We find association at variants with *P*<10^−4^ in the Ugandan cohort that are not in LD with the four highly drifted loci. The complexity of chr11p15.4 as a risk locus for haemoglobinopathies, haematological and related traits is likely because of population-specific, trait-associated variants^30^ or because of allelic heterogeneity. For example, heterogeneous signals of association in the region surrounding the causal variant for sickle cell anaemia that confers resistance to malaria have been described for three African populations, potentially because it has arisen on different haplotypes in different ancestral populations^23^,^26^. We do not find strong LD between the Pomak trait-associated variants with established variants for malaria severity and resistance^23^,^26^,^28^,^34^,^55^. However, we find that positive selection is likely to have acted on the Pomak haplotype.

Nonsynonymous variants in the human population are less frequently observed than expected based on the overall mutation rate^55^. Most alterations are deleterious and will be eventually eliminated because of purifying selection; however, beneficial mutations can sweep through the population and become fixed. We observed a significant enrichment of missense variants among loci that have drifted up in allele frequency by more than fivefold in the MANOLIS cohort. The highest fold difference in MANOLIS was observed for rs4135336. This variant is located in *PPARG*, which encodes a member of the peroxisome proliferator-activated receptor subfamily of nuclear receptors. PPARG is thought to regulate adipocyte differentiation and glucose homeostasis. Variants in *PPARG* have been associated with type 2 diabetes, obesity, atherosclerosis, cancer and hypertension^56^,^57^,^58^. Rs4135336 is located 43 kb downstream from T2D-associated SNPs (*P*<5 × 10^−8^) in the type 2 diabetes DIAGRAM meta-analysis^59^; however, these are not in LD (*r*^2^<0.2) with rs4135336. Similarly, we do not find this variant to be in LD with body mass index-associated variants from the GIANT study^60^, and rs4135336 is not associated with anthropometric or glycaemic traits in the MANOLIS cohort.

We evaluated power gains in detecting low frequency and rare variant associations afforded by studying the isolates. For an effect size of 1 s.d. (explaining 2% of trait variance for a variant with frequency of 0.01), the increase in power ranges from 0.4 to 100% with a median increase of >64%. The MCV-associated A allele of rs7116019 has increased in frequency to 0.044 in the Pomak population, empowering its discovery with genome-wide significance in a sample size of less than a thousand individuals. Ten times as many samples would be required to achieve 80% power to detect the observed effect in a nonisolated European population such as TEENAGE (risk allele frequency 0.0014).

It has been well documented that the use of genetically isolated populations can empower the identification of rare variants associated with complex traits, mainly through reduced allelic variability and genetic drift^61^. Here we have established the HELIC cohorts as population isolates through various metrics in terms of levels of isolatedness, reduced haplotype complexity and genetic drift. Leveraging these unique characteristics we show power gains that can be afforded by the study of isolated populations both theoretically and empirically by detecting variant associations with clinically important complex traits.

## Methods

### Study samples

*HELIC*. This work utilized data from 1,364 HELIC-MANOLIS and 1,883 HELIC-Pomak samples. The HELIC collections include blood for DNA extraction, laboratory-based haematological and biochemical measurements, and interview-based questionnaire data. The study was approved by the Harokopio University Bioethics Committee, and informed consent was obtained from human subjects.

*TEENAGE*. The TEENAGE (TEENs of Attica: Genes and Environment) study comprises 857 adolescents of Greek origin randomly recruited from public secondary schools located in the wider Athens area of Attica in Greece^62^.

### Genotyping and quality control

DNA samples from the MANOLIS and the Pomak collections were genotyped using Illumina HumanOmniExpress BeadChips (Illumina, San Diego, USA) at the Wellcome Trust Sanger Institute, Hinxton, UK. Genotypes of both cohorts were called together using the Illuminus genotype calling algorithm^63^ and quality control (QC) was performed separately. The initial data sets comprised 1,364 MANOLIS individuals, 1,059 Pomak individuals and 733,202 SNPs. Samples underwent standard QC procedures, with exclusion criteria as follows: (i) sample call rate <98%; (ii) samples with sex discrepancies; (iii) samples who were outliers for autosomal heterozygosity; (iv) duplicated and related samples identified by calculating the pairwise identity by descent for each sample using PLINK v1.07 (ref. 64); from each pair with a pi-hat>0.2 the sample with the lower call rate was excluded; (v) samples with evidence of non-European descent or outliers from the main cluster as assessed by MDS in PLINK^64^ by combining each population with populations from HapMap2 (release 23)^65^. SNP exclusion criteria were as follows: (i) call rate <95% (for SNPs with MAF ≥5%) or call rate <99% for SNPs with MAF <5%; (ii) Hardy–Weinberg equilibrium exact *P*<0.0001. A total of 754 unrelated individuals and 655,511 SNPs passed QC in the MANOLIS data set; 567 unrelated individuals and 643,679 SNPs passed QC in the Pomak data set. Genotyping and QC of the TEENAGE cohort were performed as above (except for genome-wide heterozygosity exclusion thresholds that were set at <32% or >35%) and have been described in detail elsewhere^66^. We further excluded SNPs with MAF<5% from all analyses except from the genetic drift analyses where a MAF threshold was not imposed (but we manually inspected calling algorithm cluster plots of rare variants for the loci of interest). The resulting data sets comprised 754 unrelated individuals and 582,214 SNPs in MANOLIS, 567 unrelated individuals and 578,677 SNPs in Pomak, 707 unrelated individuals and 586,710 SNPs in TEENAGE, of which 555,403 SNPs overlapped between the three cohorts.

### Statistical analyses

Unrelated samples (*N*=754 in MANOLIS, *N*=567 in Pomak and *N*=707 in TEENAGE cohorts) were used for all statistical analyses described herein except for trait association analyses that were performed on the full set of samples passing QC in the isolates Methods, Supplementary Methods, Supplementary Tables 11 and 12. Power calculations were performed in both unrelated and in the full set of samples (*N*=1,282 for MANOLIS and *N*=1,014 for Pomak).

### Population structure

We examined population structure by performing MDS analyses in PLINK^64^ by combining genotype data from the isolates MANOLIS, Pomak with the TEENAGE general Greek population and with populations from the 1000 Genomes Project^13^ (phase1, release_v3, 20101123). Following the data merge we took the intersection of SNPs and performed LD pruning in PLINK^64^ (with options --indep 50 5 1.25) to obtain a thinned set of data containing only independent SNPs (*N*=79,015 SNPs for the MDS performed on MANOLIS, Pomak, TEENAGE and 1000 Genomes Project populations; *N*=75,477 SNPs for the MDS performed on MANOLIS, Pomak and TEENAGE).

### Isolatedness

Fhom, Fin and ROH were calculated using PLINK^64^. The estimation of Fin is based on the observed versus the expected number of homozygous genotypes per individual (PLINK option --het). Fhom is defined as the number of observed homozygous genotypes per individual, expressed as a fraction of the number of non-missing genotypes for that individual (PLINK option --het). ROH was defined using established parameters^38^, that is, comprising a minimum of 25 consecutive homozygous SNPs over a length of 1,500 Kb with a maximum gap of 100 kb between consecutive SNPs and a minimum density of 1 SNP per 50 Kb (PLINK options --homozyg --homozyg-snp 25 --homozyg-kb 1500 --homozyg-density 50 --homozyg-gap 100).

We estimated the proportion of individuals with a surrogate parent for random regions in the genome by using the method proposed in ref. 15. We used a modification of the LRP method^15^ to identify stretches of extended haplotype sharing between individuals (Supplementary Methods, Supplementary Table 13). By examining the physical and genetic lengths of haplotype sharing within and between populations, we can also estimate the average date at which these common ancestors lived (TMRCA; T; Supplementary Methods, Supplementary Fig. 12).

### Effective population size and Fst

We estimated Ne of the three cohorts in two ways: by using estimates of haplotype sharing based on the modification of the LRP method^15^ (Supplementary Methods) and by using pairwise LD information as follows: pairwise LD was calculated as the squared correlation (*r*^2^) between genotypes for 97,885 autosomal SNPs from four random chromosomes (6, 11, 15 and 20) using PLINK^64^ (options --r2 --ld-window-kb 10,000 --ld-window-r2 0). Pairwise LD values were binned according to marker genetic distance categories (250 overlapping recombination distance classes from 0.005 to 0.25 cM, with each bin corresponding to a specific time into the past) and used to derive Ne estimates^16^,^17^,^18^ considering generation times of 25 years^67^. To avoid sampling issues, the same number (*N*=567) of individuals per population was considered. The genetic distance of pairs of markers was derived from HapMap recombination maps^65^. In all pairs of populations, for all pairs of markers, genetic distance was calculated as Fst and averaged to obtain the genetic distance of population pairs.

### Analyses of genetic drift

To examine genetic drift in the population isolates, we compared the allele frequencies of all variants in each of the two isolates against their respective allele frequency in the general Greek population in two ways: for each pairwise comparison we calculated the absolute difference and the fold difference in allele frequencies for 652,812 overlapping variants in MANOLIS versus TEENAGE, and for 642,011 variants in Pomak versus TEENAGE. We inspected genotype calling intensity plots for signals showing the strongest allele frequency differences (top 30 in each of the comparisons) and excluded badly clustering variants. We investigated the biological role of these variants and the closest gene to which they reside and examined their association with HELIC study measured traits.

### Association analyses

Association analyses in the full set of samples from the MANOLIS (*N*=1,282) and Pomak (*N*=1,014) cohorts were carried out using GEMMA v0.94 (ref. 22) to account for relatedness (kinship matrix options: -bfile -gk -n -o; association test options: -bfile -n -notsnp -maf -miss -km -k -lmm -o). GEMMA fits a standard linear mixed model that accounts for sample substructure for single marker association tests, thus correcting for relatedness. This is achieved via a relatedness or kinship matrix, which is estimated from the samples’ genotypes. The centred kinship matrix was created using only variants with MAF >0.01 and missingness <0.05. We report *P* values from the score test but note that these were very similar to *P* values from the Wald and likelihood ratio tests. Details of the phenotype transformation protocol for each trait tested in each population are given in Supplementary Methods and Supplementary Tables 11 and 12.

### Conditional association analyses

Conditional association analyses were performed in GEMMA^22^ by including variant genotypes as covariates in the analysis. The most highly MCV-associated variant was tested conditioning on each of the highly drifted variants to determine whether multiple signals were present at the locus. We also conditioned on published signals at variants that were present on our array and had *r*^2^>0.2 with the most highly associated variant.

### Enrichment of functional variants

We investigated enrichment of putatively functional variants among those variants that have increased in allele frequency in the isolates compared with the general population. We annotated all variants in the data set using the Ensembl Variant Effect Predictor (version 74) and focused on variants lying in coding sequence, specifically variants called as missense, synonymous, nonsense, frameshift or overlapping an essential splice site. We found insufficient numbers of variants falling into each of the latter three categories; therefore, we limited further analysis to missense and synonymous variants only. To test for enrichment of missense and synonymous variants we used incrementally increasing thresholds of the fold change in allele frequency (1 × , 5 × , 10 × , 15 × , 20 × and 25 × ), and at each threshold we computed a contingency table comparing the number of missense and other variants found at a fold change higher than the threshold. We focused this analysis on fold changes in order to primarily capture relevant changes in allele frequency for low frequency and rare variants. We used odds ratios to quantify enrichment and Fisher’s exact tests to compute the significance of the result.

### Inbreeding depression

Linear regression was used to examine association of Fhom and F_ROH^49^ with height and with cardiometabolic traits (Supplementary Tables 11 and 12) from the HELIC and TEENAGE studies. F_ROH is the sum of the lengths of all ROH longer than 1,500 kb expressed as a percentage of the typed autosomal genome (that is, the sum of the length of all the autosomes from the first to the last SNP, excluding the centromeres)^38^,^49^.

## Additional information

**How to cite this article:** Panoutsopoulou, K. *et al*. Genetic characterization of Greek population isolates reveals strong genetic drift at missense and trait-associated variants. *Nat. Commun.* 5:5345 doi: 10.1038/ncomms6345 (2014).

**Accession codes:** The HELIC-MANOLIS genotype data (Illumina Human OmniExpress) have been deposited in the European Genome-phenome Archive (EGA) under the accession code EGAS00001000687.

## Supplementary Material

Supplementary Information Supplementary Figures 1-12, Supplementary Tables 1-13, Supplementary Notes 1-4, Supplementary Methods and Supplementary References

## Figures and Tables

**Table 1:** Allele frequency increases in the isolates, MANOLIS and Pomak, compared with the general Greek population, TEENAGE.

Fold allele frequency increase	**MANOLIS versus TEENAGE** ***N*** **(%)**	**Pomak versus TEENAGE** ***N*** **(%)**
>50–60	—	2 (0.0003)
>40–50	3 (0.0005)	2 (0.0003)
>30–40	17 (0.0026)	12 (0.0019)
>20–30	63 (0.0097)	50 (0.0078)
>10–20	434 (0.07)	195 (0.03)
>1–10	317,911 (48.70)	308,266 (48.02)

**Table 2:** Association summary statistics (score test in GEMMA) of rs7116019 with haematological traits in the Pomak population.

**SNP**	**Trait**	**EA**	Pomak discovery, *N*=1,014				Pomak replication, *N*=719				Pomak discovery and replication, *N*=1,684			
			**EAF**	**Beta**	**s.e.**	***P*** **value**	**EAF**	**Beta**	**s.e.**	***P*** **value**	**EAF**	**Beta**	**s.e.**	***P*** **value**
rs7116019	MCV	G	0.047	−1.239	0.105	2.3 × 10^−26^	0.030	−1.220	0.147	1.6 × 10^−14^	0.039	−1.210	0.088	2.0 × 10^−36^
rs7116019	MCHC	G	0.047	0.933	0.107	7.1 × 10^−16^	0.029	1.219	0.146	8.6 × 10^−15^	0.040	1.010	0.088	8.3 × 10^−27^
rs7116019	MCH	G	0.046	−0.775	0.111	1.8 × 10^−11^	0.029	−0.582	0.154	1.2 × 10^−04^	0.039	−0.702	0.092	1.7 × 10^−13^

EA, effect allele; EAF, effect allele frequency; MCH, mean corpuscular haemoglobin; MCHC, mean corpuscular haemoglobin concentration; MCV, mean corpuscular volume; SNP, single-nucleotide polymorphism.
