# Supplementary material for: Genetic characterization of Greek population isolates reveals strong genetic drift at missense and trait-associated variants
Source: Nat Commun. 2014 Nov 6;5:5345. doi: 10.1038/ncomms6345 (PMC4242463; doi:10.1038/ncomms6345)
Supplement: Supplementary Information — Supplementary Figures 1-12, Supplementary Tables 1-13, Supplementary Notes 1-4, Supplementary Methods and Supplementary References [file ncomms6345-s1.pdf]

Supplementary Figures

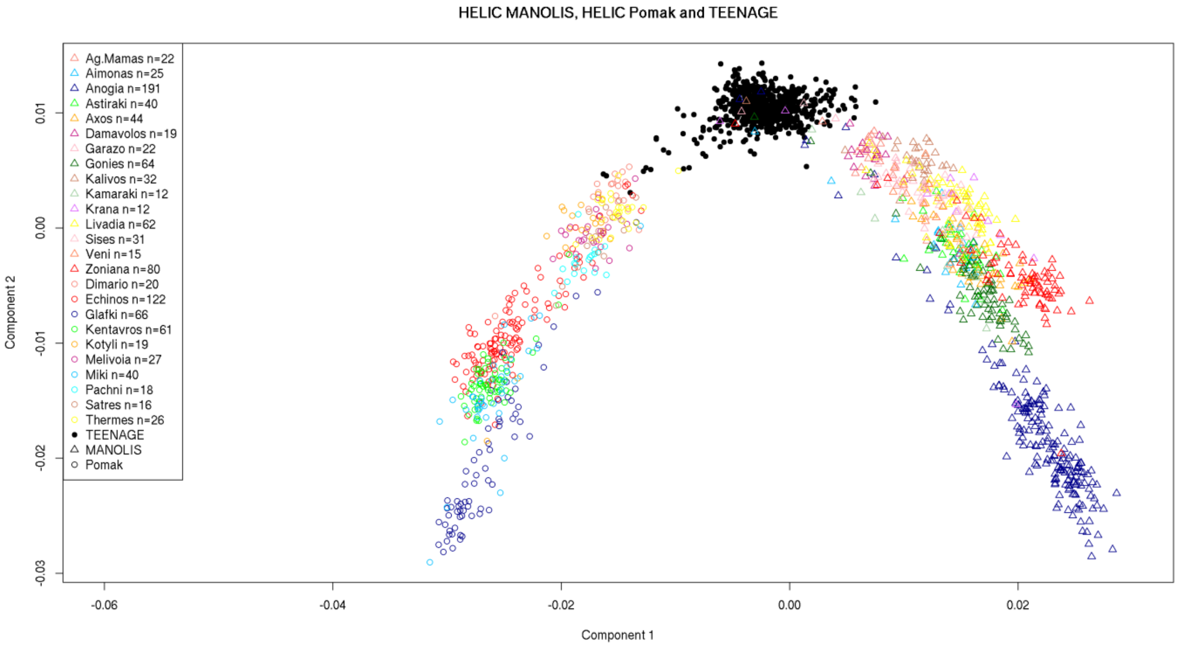

Supplementary Figure 1. Multidimensional scaling (MDS) analysis of TEENAGE, HELIC-MANOLIS villages and HELIC-Pomak villages carried out with a subset of Kentavros individuals. The black solid circles depict individuals from TEENAGE representing the general Greek population. Individuals from the MANOLIS cohort are depicted by the differently coloured hollow triangles with each colour corresponding to the village of origin. Individuals from the Pomak villages are depicted by the differently coloured hollow circles with each colour corresponding to the village of origin. Here the sample size of Kentavros (N=61) is comparable to the sample size of the other Pomak villages and does not form a separated cluster as in Figure 1.

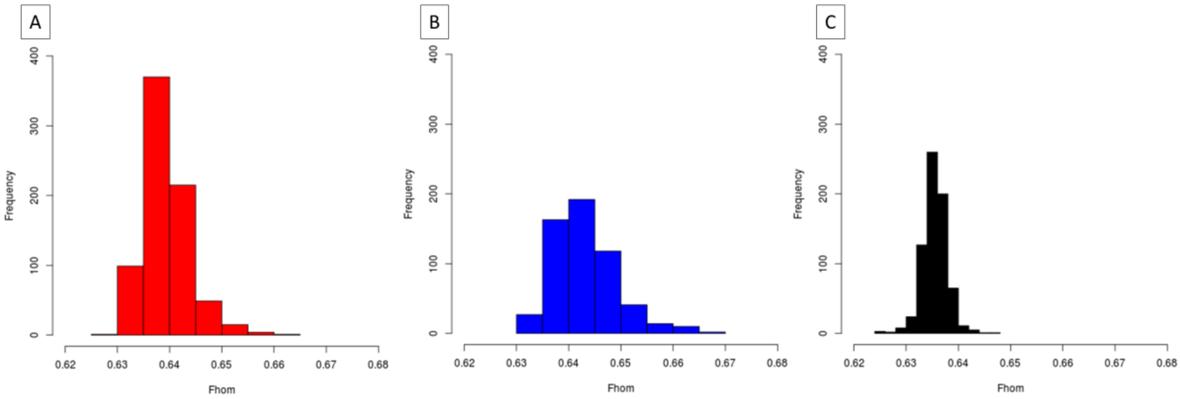

Supplementary Figure 2. Distribution of genome-wide homozygosity (Fhom). (A) MANOLIS, (B) Pomak and (C) TEENAGE.

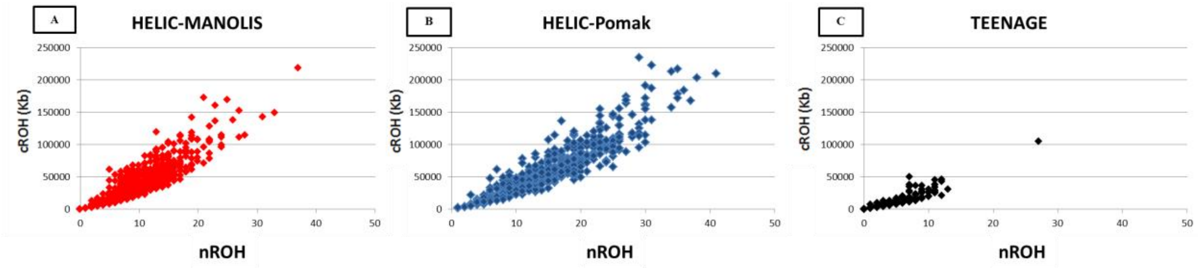

Supplementary Figure 3. Cumulative length of ROHs (cROH) plotted against number of ROHs (nROHs). (A) MANOLIS, (B) Pomak and (C) TEENAGE.

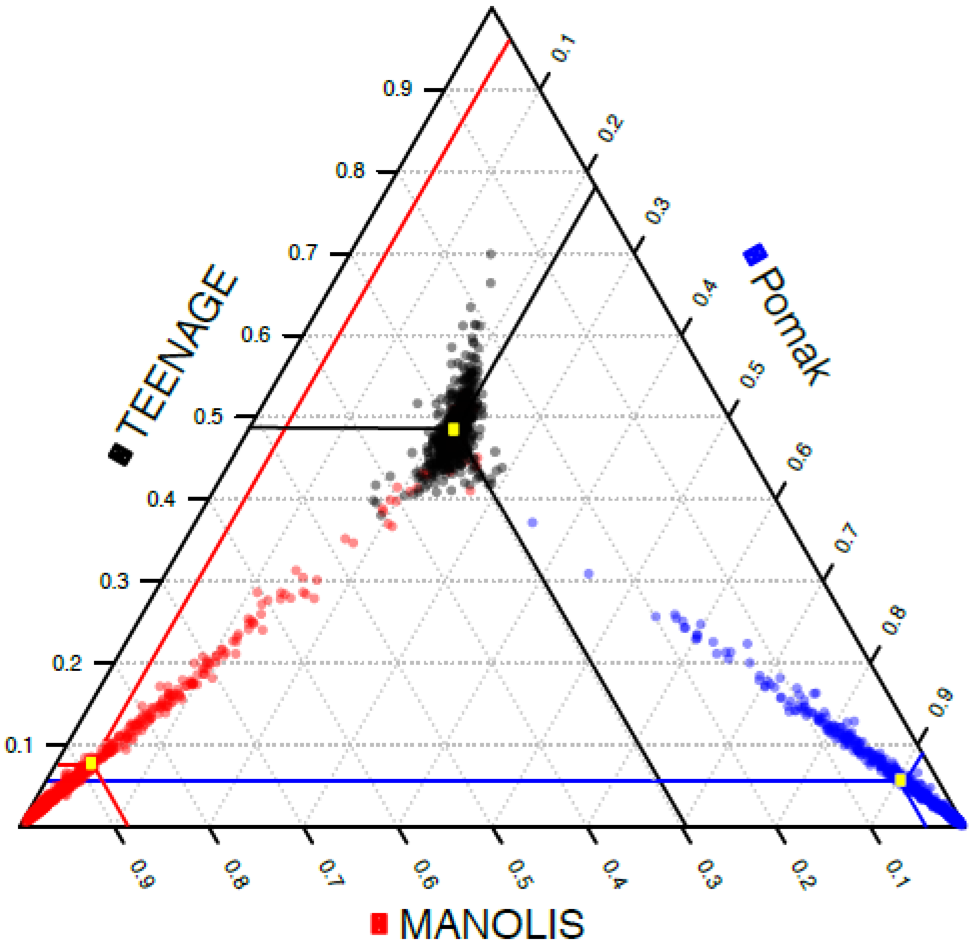

Supplementary Figure 4. Choosing nearest neighbours (NNs) across cohorts. The triangle plot shows how often individuals in each cohort (MANOLIS, red; Pomak, blue; TEENAGE, black) select NNs from each of the three cohorts when the algorithm was run with all three cohorts lumped together. The yellow points show the mean values for each cohort. For example, a MANOLIS individual has NNs that are also MANOLIS 88.3% of the time, NNs from the Pomaks 3.8% and TEENAGE 7.8% of the time. The results are genome-wide means per person.

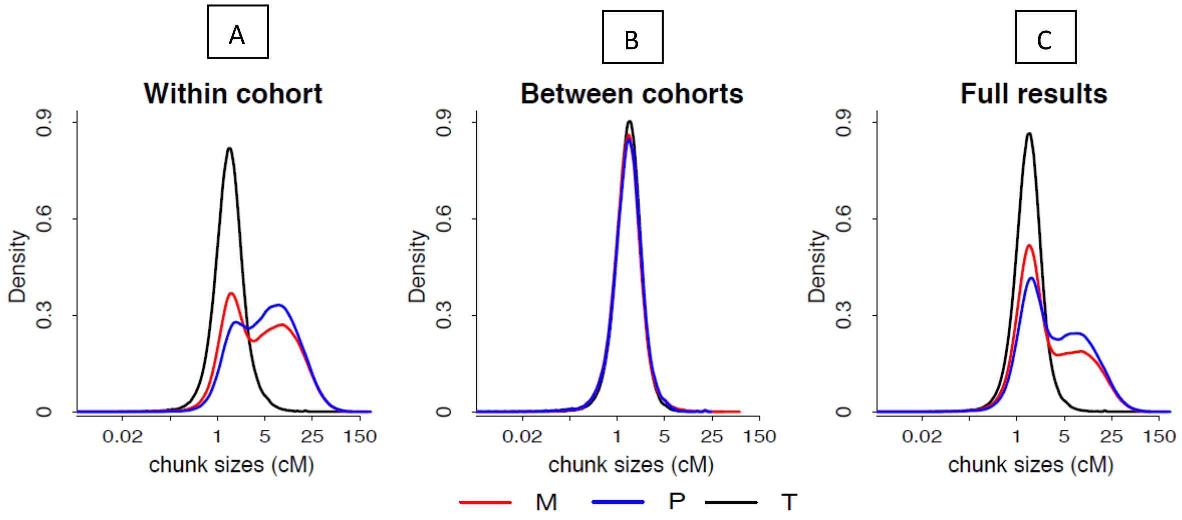

Supplementary Figure 5. Density curves for the haplotype chunks when the algorithm is run with all samples lumped together. Panel A shows the density of chunks shared with nearest neighbours (NNs) that belong to the same cohort as the query individual (red, M = MANOLIS; blue, P = Pomaks; black, T = TEENAGE). Panel B looks at chunks for the cases where an individual's NN is from a different cohort: red = between MANOLIS and TEENAGE; blue = between Pomaks and Teenage; black = between TEENAGE and either MANOLIS or Pomaks. Panel C looks at the full density of shared chunk sizes with all of an individual's NNs, independently of the NNs' cohort. The results have been combined across chromosomes as there was minimal variation among them.

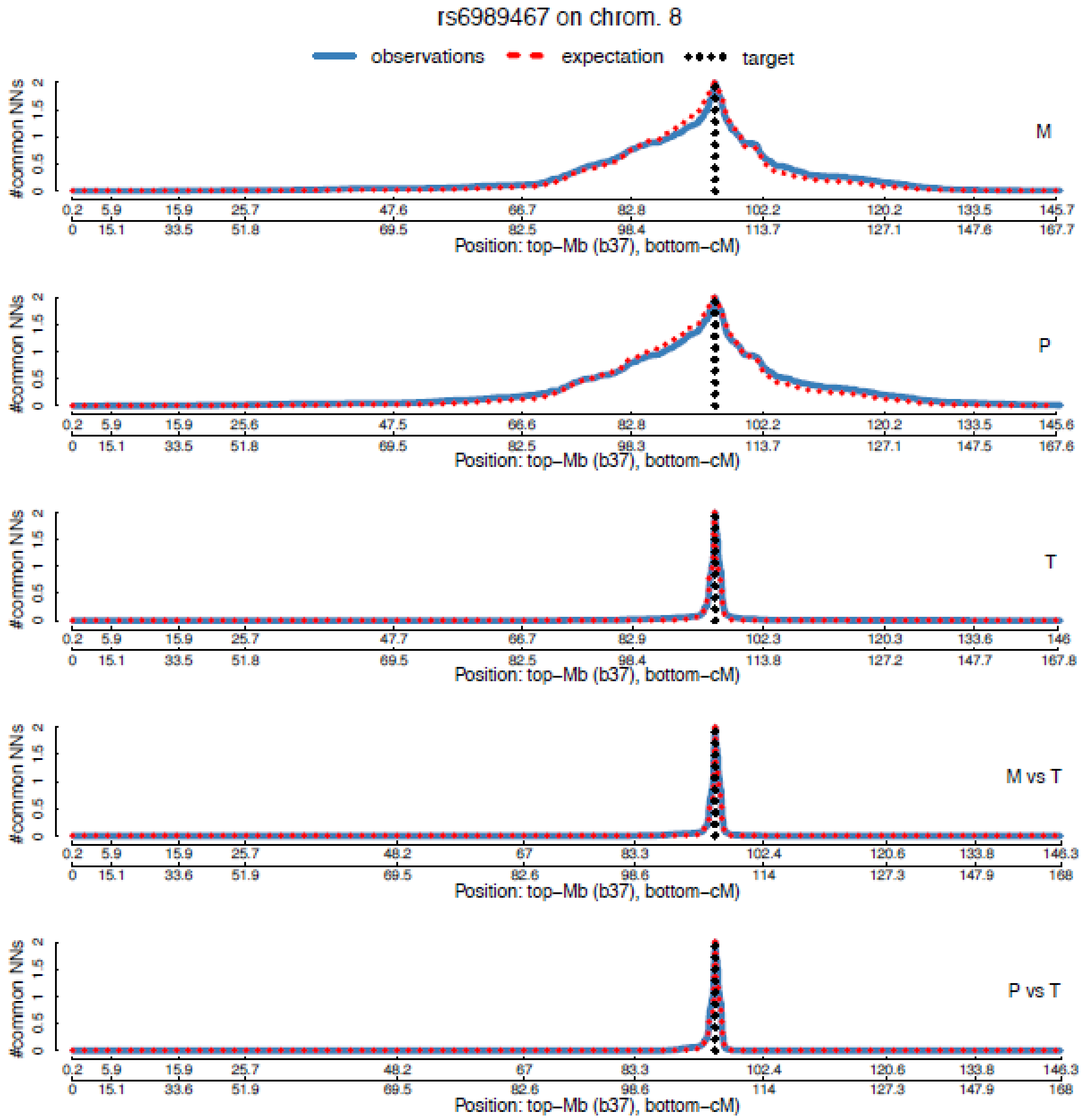

Supplementary Figure 6. The decay of haplotype sharing with an individual's nearest neighbours (NNs) at rs6989467 (randomly selected) on chromosome 8. The x-axis is position on chromosome 8 (physical, Mb and genetic, cM) and the y axis is the number of NNs that are unchanged (compared against the NN choice at rs6989467), averaged over all the individuals in the sample. In blue is the observation for each cohort and in red the expected curve of NN sharing decay (see Methods). The top 3 plots show results when the algorithm was run on each cohort (MANOLIS = M; Pomak = P; TEENAGE = T) separately. At rs6989467, the mean TMRCA with a NN among the MANOLIS individuals is 7.3 generations, among the Pomaks it is 6.4 generations, while among the TEENAGE cohort it is 77.2 generations. The bottom two plots show results for the MANOLIS (M vs T) and for the Pomak (P vs T) individuals when forced to pick NNs from the TEENAGE cohort. In the former case the mean TMRCA to a TEENAGE NN is 100.4 generations (at this SNP) and in the latter it is 89.2 generations.

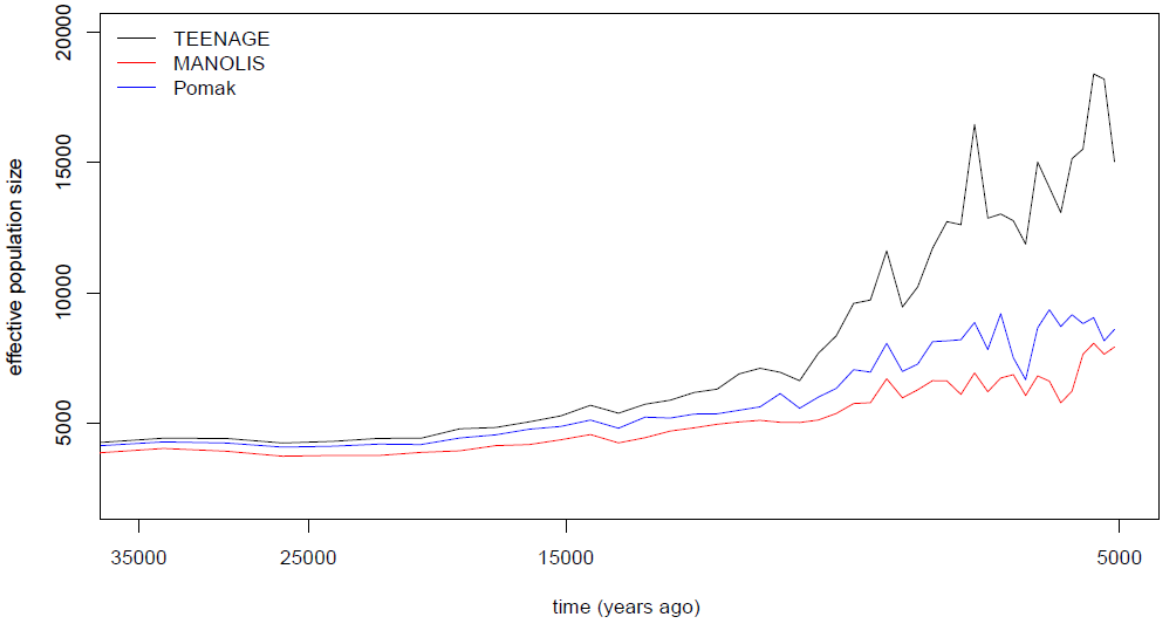

Supplementary Figure 7. Different trends of effective population size (Ne) through time between the two isolated populations, MANOLIS and Pomak, and the outbred TEENAGE population.

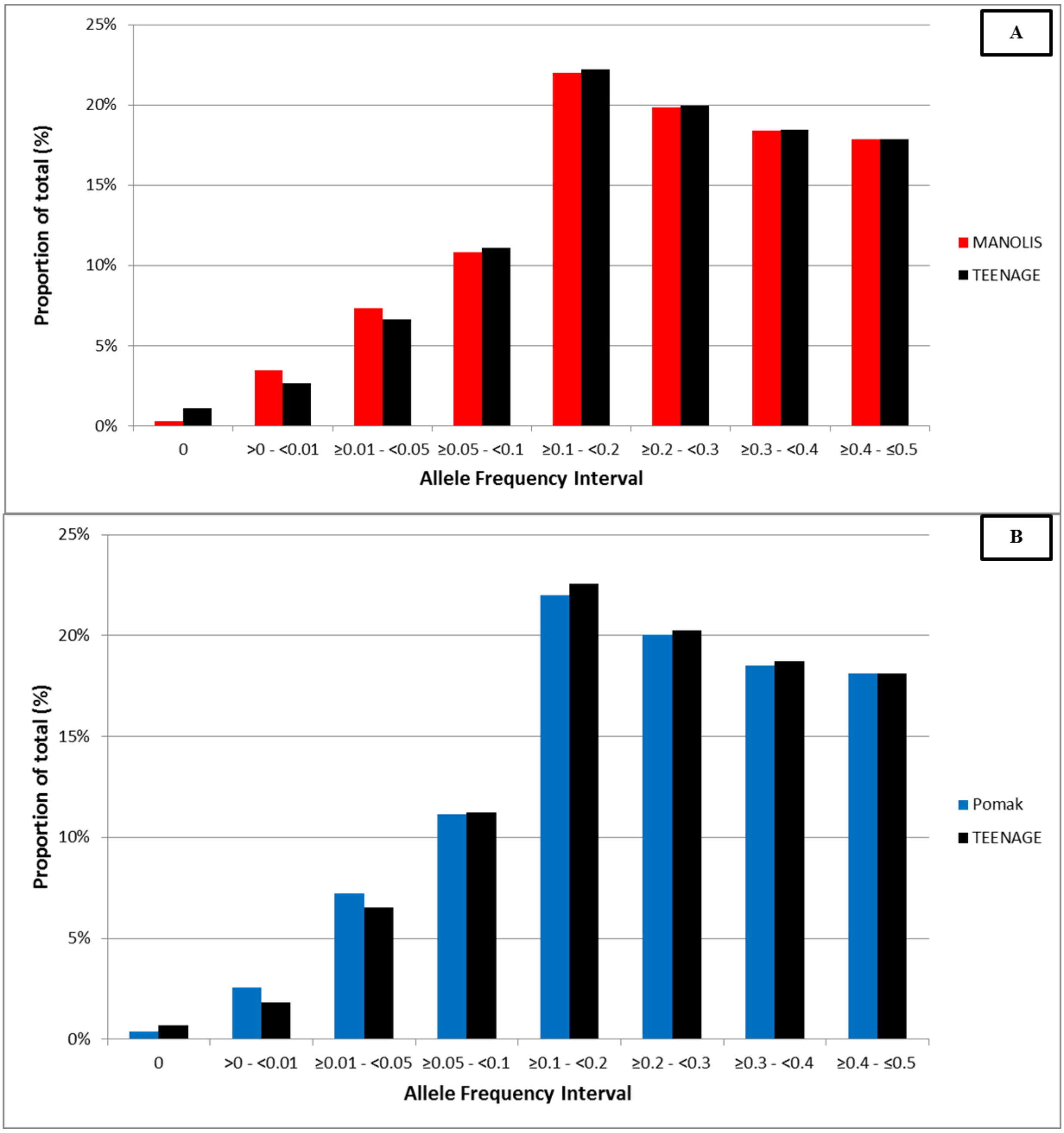

Supplementary Figure 8. Allele frequency distribution for overlapping variants between (A) MANOLIS and TEENAGE and (B) Pomak and TEENAGE.

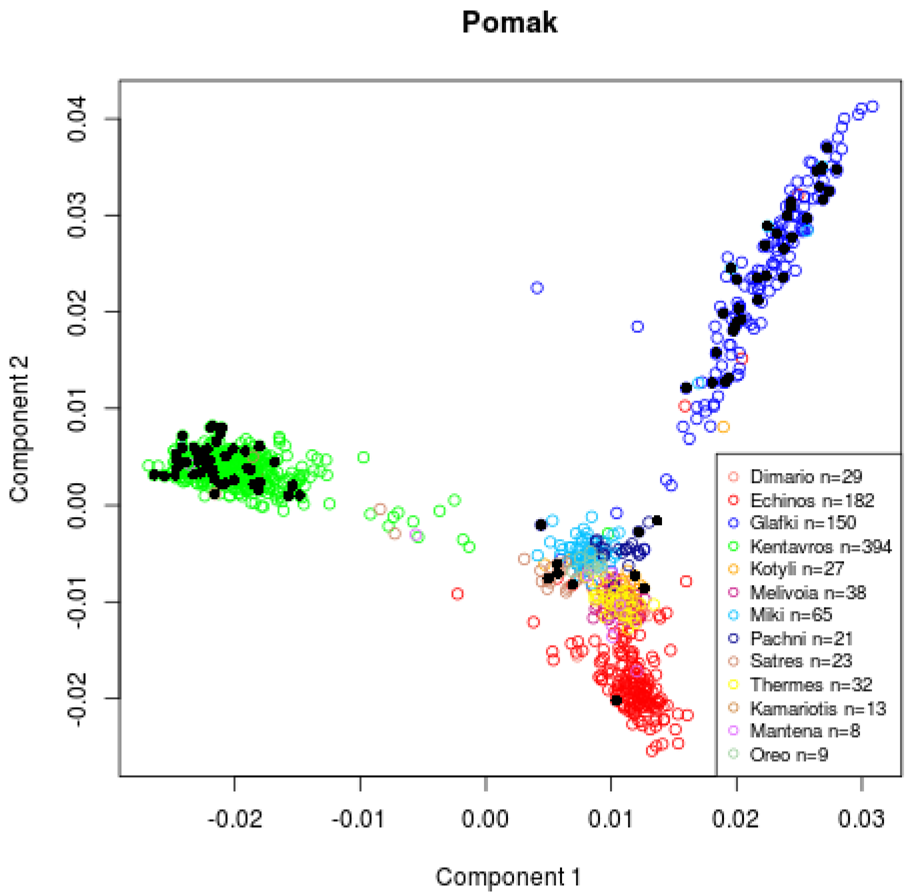

Supplementary Figure 9. Multidimensional scaling (MDS) analysis plot showing village of origin for minor allele carriers at rs7116019 in the Pomak cohort. Heterozygotes and homozygotes for the minor allele at rs7116019 (GA=87; GG=5) are coloured black.

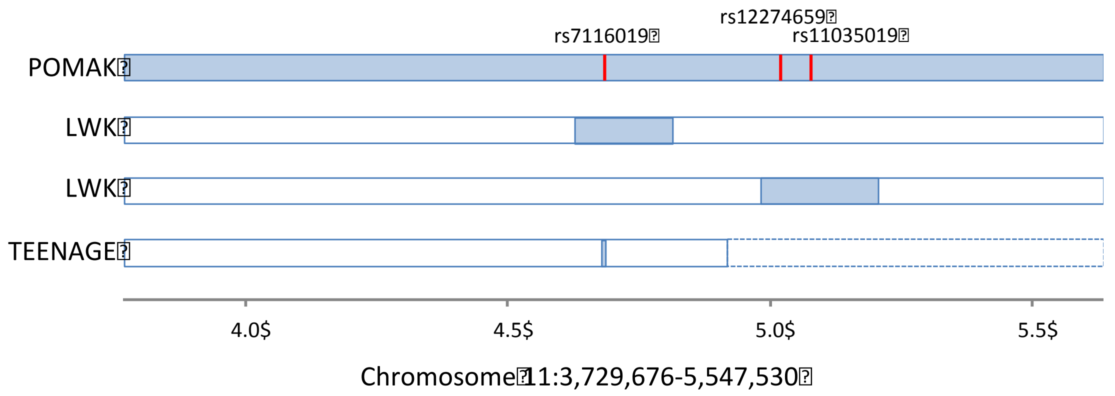

Supplementary Figure 10. The shared haplotype block containing rs7116019G, rs12274659G and rs11035019T among Pomak, TEENAGE, and the Luhya (LWK) population from the 1000 Genomes Project. Shaded regions represent haplotype blocks shared within the Pomak (top) or between Pomak and other populations (lower three sections). The region outlined by the dashed line has no information.

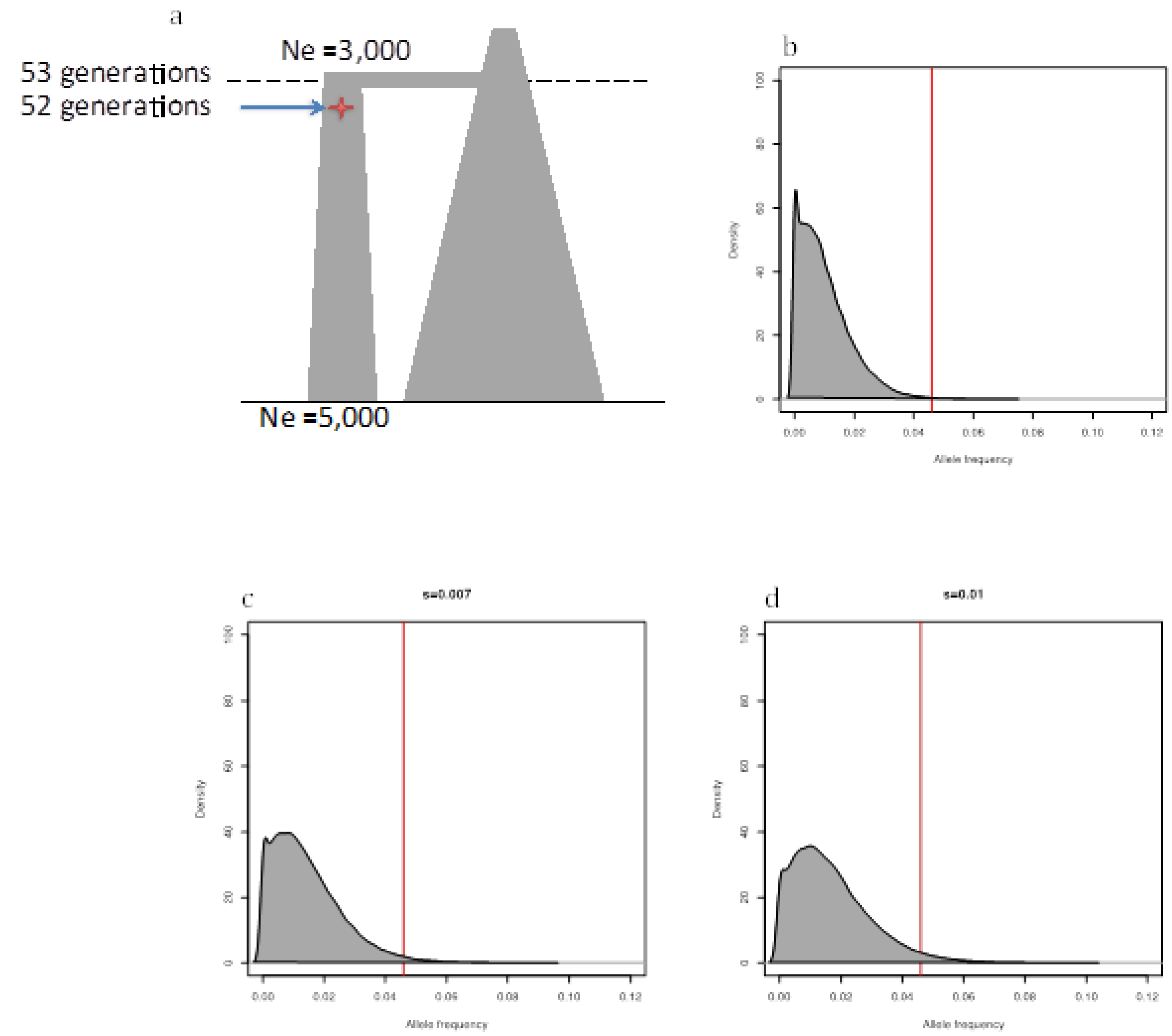

Supplementary Figure 11. Demographic model used and distribution of allele frequencies in simulations under neutrality or positive selection. a: demographic model. b: distribution of allele frequencies for one locus after 52 generations of neutral drift under this demographic model. c, d: distribution of allele frequencies for one locus after 52 generations of positive selection with selection coefficient s =0.007 and s=0.01. The vertical red line corresponds to the observed value in the Pomak isolate.

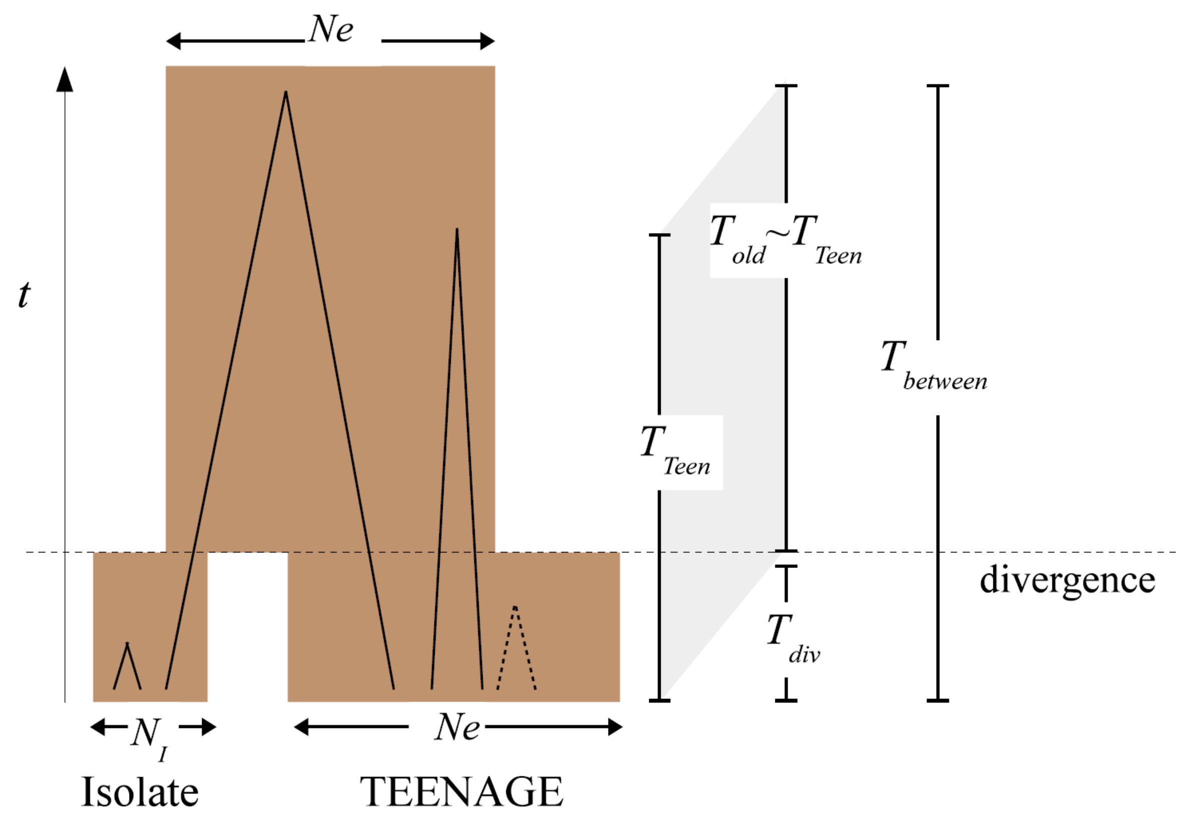

Supplementary Figure 12. Estimating time of divergence. The figure demonstrates the event of divergence (horizontal dashed line) between an isolate and the TEENAGE cohort from an ancestral population. Tdiv is the time at which divergence took place. The brown shaded region represents the genealogy, and includes possible coalescence events marked by the black lines. The dotted black lines showing a recent coalescence between TEENAGE nearest neighbours (NNs) before the time of divergence highlight the type of events that we are assuming have not taken place. The width of each part of the genealogy is proportional to the effective population size, which is NI for the isolate and Ne for the TEENAGE cohort and ancestral population. In other words, we are further assuming here that the effective population size for the TEENAGE cohort does not change going backwards in time. In this set up we may calculate the TMRCA between TEENAGE NNs, Tteen, (by picking NNs only from within the isolate cohort) and the TMRCA between NNs when these involve an individual from an isolate and an individual from the TEENAGE samples, Tbetween. Told is Tteen − Tdiv, the time to the TMRCA between TEENAGE individuals since divergence. We approximate Told by Tteen. By examining the physical and genetic lengths of haplotype sharing within and between populations, we can also estimate the average date at which these common ancestors lived (TMRCA; T) (Supplementary Methods).

Supplementary Tables

Supplementary Table 1. Pairwise Fst values between the isolated populations (MANOLIS and Pomak) and the outbred Greek population (TEENAGE) calculated for random chromosomes.

Pairwise Fst	chr6	chr11	chr15	chr20
MANOLIS vs TEENAGE	0.0022	0.0022	0.0023	0.0022
Pomak vs TEENAGE	0.0032	0.0032	0.0034	0.0031
MANOLIS vs Pomak	0.0053	0.0049	0.0058	0.0051

Supplementary Table 2. Inbreeding coefficient (Fin), number and cumulative length of ROH (nROH and cROH, respectively) in the two isolates, MANOLIS and Pomak, and in the non-isolated Greek TEENAGE population.

	Fin<br>Mean (SD)	nROH<br>Mean (SD)	cROH(kb)<br>Mean (SD)	nROH_LD<br>Mean (SD)	cROH_LD(kb)<br>Mean (SD)
MANOLIS	0.007 (0.121)	9.3 (5.1)	37393 (29041)	1.7 (2.0)	3607 (4196)
Pomak	0.014 (0.017)	15.4 (6.9)	62608 (39356)	3.0 (2.7)	6278 (5743)
TEENAGE	0.0004 (0.007)	4.0 (2.3)	8381 (7249)	0.1 (0.4)	194 (755)

Supplementary Table 3. Allele frequency differences in the MANOLIS vs TEENAGE and Pomak vs TEENAGE analyses binned according to allele frequency in TEENAGE.
AF, Allele frequency

AF in TEENAGE	N (%) variants increased	N (%) variants decreased	N (%) variants unchanged	Mean absolute AF increase	Mean absolute AF decrease	Mean fold AF increase	Mean fold AF decrease
MANOLIS vs TEENAGE							
0	6261 (0.96)	0 (0.00)	957 (0.15)	0.0061	NA	NA	NA
0-0.01	9083 (1.39)	8197 (1.26)	0 (0.00)	0.0064	-0.0023	3.61	2.71
0.01-0.05	20820 (3.19)	22385 (3.43)	17 (0.003)	0.0145	-0.0101	1.52	1.92
0.05-0.1	35298 (5.41)	37311 (5.72)	5 (0.001)	0.0207	-0.0172	1.28	1.38
0.1-0.2	71101 (10.89)	73907 (11.32)	180 (0.03)	0.0271	-0.0239	1.19	1.23
0.2-0.3	64466 (9.88)	65711 (10.07)	148 (0.02)	0.0324	-0.0299	1.13	1.15
0.3-0.4	59555 (9.12)	60848 (9.32)	102 (0.02)	0.0353	-0.0337	1.10	1.12
0.4-0.5	58105 (8.90)	58264 (8.93)	91 (0.01)	0.0363	-0.0356	1.08	1.09
Pomak vs TEENAGE							
0	3395 (0.53)	0 (0.00)	1048 (0.16)	0.0075	NA	NA	NA
0-0.01	5514 (0.86)	6154 (0.96)	0 (0.00)	0.0074	-0.0031	3.46	3.17
0.01-0.05	19486 (3.04)	22432 (3.49)	3 (0.0005)	0.0184	-0.0122	1.64	2.54
0.05-0.1	34185 (5.32)	37943 (5.91)	16 (0.002)	0.0259	-0.0209	1.35	1.53
0.1-0.2	69304 (10.79)	75507 (11.76)	121 (0.02)	0.0340	-0.0299	1.23	1.31
0.2-0.3	63496 (9.89)	66568 (10.37)	104 (0.02)	0.0404	-0.0377	1.16	1.20
0.3-0.4	59031 (9.19)	61282 (9.55)	75 (0.01)	0.0439	-0.0423	1.13	1.15
0.4-0.5	57511 (8.96)	58743 (9.15)	93 (0.01)	0.0453	-0.0444	1.10	1.12

Supplementary Table 4. Association summary statistics for variants associated with genome-wide significance with mean corpuscular volume (MCV) in the Pomak cohort and their respective summary statistics in the General Population Cohort (GPC). MCV in the Pomak cohort was inverse-normalised, then z-standardised. MCV in the GPC cohort was inverse-normalised and adjusted for age, age^2 and sex.
EA, effect allele; NEA, Non-effect allele; EAF, Effect allele frequency

**MCV SNPs**			**Pomak**						**General Population Cohort**					
**Chr**	**SNP**	**bp**	**EA**	**NEA**	**EAF**	**BETA**	**SE**	**p value**	**EA**	**NEA**	**EAF**	**BETA**	**SE**	**p value**
11	rs12274659	4947444	G	A	0.051	-1.249	0.099	3.45E-29	G	A	0.151	0.020	0.053	7.06E-01
11	rs11035019	5009563	T	C	0.051	-1.249	0.099	3.45E-29	T	C	0.063	0.067	0.084	4.22E-01
11	rs16906901	4901939	G	A	0.051	-1.211	0.099	1.15E-27	G	A	0.180	-0.083	0.051	1.00E-01
11	rs16911934	5251478	C	T	0.056	-1.144	0.096	1.80E-26	C	T	0.280	-0.008	0.042	8.53E-01
11	rs16911998	5257700	A	G	0.056	-1.143	0.096	1.96E-26	A	G	0.217	0.019	0.046	6.80E-01
11	rs16924612	4669708	T	C	0.047	-1.240	0.105	2.18E-26	T	C	0.240	-0.004	0.045	9.33E-01
11	rs7116019	4618606	G	A	0.047	-1.239	0.105	2.26E-26	G	A	0.156	-0.023	0.054	6.66E-01
11	rs7929591	5150372	C	T	0.055	-1.108	0.096	1.20E-25	C	T	0.227	-0.047	0.045	2.98E-01
11	rs2133233	5466043	T	C	0.056	-1.077	0.095	2.22E-24	T	C	0.071	-0.042	0.074	5.71E-01
11	rs16929597	4238136	A	G	0.047	-1.183	0.108	1.07E-23	A	G	0.101	-0.064	0.063	3.14E-01
11	rs16929572	4238237	C	T	0.047	-1.183	0.108	1.07E-23	C	T	0.151	-0.031	0.054	5.64E-01

11	rs6578634	5420909	G	T	0.067	-0.962	0.089	8.99E-23	G	T	0.091	-0.088	0.067	1.87E-01
11	rs7122404	6014364	T	G	0.053	-1.065	0.102	1.00E-21	T	G	0.258	-0.004	0.044	9.25E-01
11	rs2167562	3970371	T	C	0.049	-1.095	0.106	1.56E-21	T	C	0.165	0.104	0.051	4.11E-02
11	rs16913631	6467984	A	C	0.051	-1.046	0.106	6.86E-20	A	C	0.111	-0.064	0.060	2.90E-01
11	rs12226204	6189514	G	A	0.061	-0.903	0.095	2.05E-18	G	A	0.171	-0.040	0.051	4.34E-01
11	rs11033308	4702108	T	C	0.067	-0.845	0.090	4.12E-18	T	C	0.103	-0.014	0.062	8.17E-01
11	rs12223580	4706179	A	G	0.067	-0.845	0.090	4.40E-18	A	G	0.101	0.018	0.062	7.74E-01
11	rs1124113	4700862	T	C	0.079	-0.755	0.084	5.26E-17	T	C	0.121	-0.013	0.059	8.26E-01
11	rs6578839	7260691	T	C	0.055	-0.893	0.103	5.13E-16	T	C	0.390	0.039	0.039	3.17E-01
11	rs4992800	5702462	A	C	0.074	-0.744	0.088	1.19E-15	A	C	0.333	0.017	0.040	6.76E-01
11	rs16908782	5054643	C	T	0.160	-0.532	0.063	2.05E-15	C	T	0.109	0.091	0.058	1.16E-01
11	rs16908907	5057550	G	A	0.160	-0.532	0.063	2.05E-15	G	A	0.073	0.084	0.070	2.33E-01
11	rs7951380	4852462	A	G	0.097	-0.630	0.075	2.21E-15	A	G	0.174	-0.014	0.047	7.66E-01
11	rs16906747	4855124	C	A	0.098	-0.630	0.075	2.23E-15	C	A	0.224	0.007	0.044	8.78E-01
11	rs7108599	4607913	A	G	0.092	-0.633	0.077	6.75E-15	A	G	0.166	-0.036	0.051	4.77E-01

11	rs11602279	5209592	A	G	0.101	-0.609	0.074	9.03E-15	G	A	0.477	-0.034	0.038	3.68E-01
11	rs3861787	4647770	T	G	0.094	-0.625	0.077	1.14E-14	T	G	0.460	-0.022	0.039	5.62E-01
11	rs3863292	5318677	T	C	0.106	-0.606	0.074	2.02E-14	T	C	0.159	-0.027	0.052	6.07E-01
11	rs1552726	7071031	G	A	0.042	-0.966	0.121	5.14E-14	G	A	0.262	-0.020	0.042	6.30E-01
11	rs7108834	5662319	C	T	0.093	-0.633	0.078	5.14E-14	C	T	0.193	-0.091	0.049	6.25E-02
11	rs7109861	5664974	T	C	0.093	-0.632	0.078	5.56E-14	T	C	0.371	-0.015	0.038	6.94E-01
11	rs7106798	5665225	A	G	0.093	-0.632	0.078	5.56E-14	A	G	0.371	-0.014	0.038	7.11E-01
11	rs7950390	4631352	G	T	0.100	-0.587	0.075	6.27E-14	G	T	0.466	0.040	0.038	2.91E-01
11	rs16931173	5469959	C	A	0.119	-0.562	0.072	2.94E-13	C	A	0.157	0.039	0.052	4.48E-01
11	rs766498	7257474	T	C	0.064	-0.740	0.096	3.20E-13	T	C	0.418	0.026	0.038	4.90E-01
11	rs12226152	5652341	A	G	0.097	-0.587	0.076	3.66E-13	A	G	0.184	-0.079	0.050	1.17E-01
11	rs904376	5652608	G	T	0.127	-0.498	0.067	2.98E-12	G	T	0.238	-0.109	0.045	1.50E-02
11	rs885002	5710183	A	G	0.084	-0.591	0.083	8.87E-12	A	G	0.154	0.006	0.052	9.13E-01
11	rs12271916	4726878	A	G	0.113	-0.518	0.073	9.20E-12	A	G	0.450	0.017	0.038	6.63E-01
11	rs4459298	4596449	A	G	0.153	-0.451	0.064	1.22E-11	A	G	0.349	-0.023	0.040	5.58E-01

11	rs17238860	6242337	C	T	0.091	-0.575	0.081	1.84E-11	C	T	0.058	0.058	0.082	4.79E-01
11	rs16907494	4953022	C	T	0.195	-0.401	0.058	2.87E-11	C	T	0.175	0.061	0.049	2.09E-01
11	rs1123991	4703165	C	A	0.166	-0.428	0.062	5.40E-11	A	C	0.480	0.052	0.037	1.58E-01
11	rs12577475	4913314	A	G	0.134	-0.474	0.069	5.88E-11	A	G	0.129	0.054	0.057	3.45E-01
11	rs10836914	4913057	G	A	0.134	-0.472	0.069	7.38E-11	G	A	0.198	0.024	0.047	6.08E-01
11	rs2445311	5089017	C	T	0.218	-0.380	0.056	7.45E-11	C	T	0.288	0.033	0.042	4.35E-01
11	rs7110328	4500876	A	G	0.103	-0.508	0.076	1.68E-10	G	A	0.431	0.034	0.038	3.69E-01
11	rs2857919	7022160	A	G	0.062	-0.663	0.099	1.90E-10	A	G	0.276	0.000	0.042	9.91E-01
11	rs1352860	7020687	C	T	0.062	-0.663	0.099	1.97E-10	C	T	0.277	0.001	0.042	9.80E-01
11	rs16907439	4946056	T	C	0.147	-0.420	0.065	5.01E-10	T	C	0.094	0.075	0.066	2.55E-01
11	rs1388526	7020629	A	G	0.056	-0.675	0.104	7.83E-10	A	G	0.269	0.011	0.043	7.91E-01
11	rs1805002	6291120	A	G	0.102	-0.500	0.079	8.92E-10	A	G	0.048	0.032	0.088	7.17E-01
11	rs7126152	6705253	A	G	0.090	-0.519	0.082	9.38E-10	G	A	0.319	-0.059	0.041	1.44E-01
11	rs11034573	4919979	G	A	0.150	-0.426	0.067	1.12E-09	G	A	0.191	0.014	0.049	7.75E-01
11	rs12296090	6705464	C	A	0.090	-0.519	0.083	1.33E-09	A	C	0.325	-0.079	0.041	5.18E-02

11	rs11036212	5221825	G	A	0.150	-0.405	0.065	1.43E-09	G	A	0.433	0.073	0.039	6.30E-02
11	rs7128249	6700887	C	T	0.090	-0.511	0.082	1.57E-09	T	C	0.498	-0.022	0.038	5.61E-01
11	rs7129059	4587940	A	G	0.108	-0.466	0.074	2.16E-09	A	G	0.409	-0.022	0.039	5.73E-01
11	rs4573672	4596465	T	C	0.189	-0.362	0.059	2.72E-09	T	C	0.447	-0.030	0.038	4.27E-01
11	rs2709159	4538034	C	T	0.258	-0.316	0.054	1.05E-08	C	T	0.135	-0.050	0.054	3.53E-01
11	rs2709193	4527196	T	C	0.254	-0.313	0.054	1.53E-08	T	C	0.075	0.034	0.069	6.26E-01
11	rs2566238	4533118	A	G	0.254	-0.313	0.054	1.53E-08	A	G	0.074	0.041	0.069	5.49E-01
11	rs10839547	6320703	A	G	0.146	-0.391	0.067	1.60E-08	A	G	0.152	-0.115	0.051	2.62E-02
11	rs4910716	3624237	G	A	0.095	-0.438	0.076	2.07E-08	G	A	0.112	0.004	0.060	9.50E-01
11	rs4269953	5357881	C	T	0.147	-0.375	0.065	2.31E-08	T	C	0.405	-0.005	0.038	9.05E-01
11	rs4758408	6348639	T	C	0.139	-0.391	0.068	2.54E-08	C	T	0.420	-0.004	0.039	9.17E-01
11	rs1488864	6342329	T	G	0.140	-0.390	0.068	2.62E-08	G	T	0.479	-0.014	0.039	7.24E-01
11	rs7105981	4980153	G	A	0.214	-0.319	0.056	4.85E-08	G	A	0.254	0.015	0.043	7.27E-01

Supplementary Table 5. Association summary statistics for variants associated with genome-wide significance with mean corpuscular haemoglobin concentration (MCHC) in the Pomak cohort and their respective summary statistics in the General Population Cohort (GPC). MCHC in the Pomak cohort was untransformed, adjusted for age and age^2, then z-standardised. MCHC in the GPC cohort was inverse-normalised and adjusted for age, age^2 and sex.
EA, effect allele; NEA, Non-effect allele; EAF, Effect allele frequency

MCHC SNPs			Pomak						General Population Cohort					
**Chr**	**SNP**	**Bp**	**EA**	**NEA**	**EAF**	**BETA**	**SE**	**p value**	**EA**	**NEA**	**EAF**	**BETA**	**SE**	**p value**
11	rs12274659	4947444	G	A	0.052	1.010	0.100	5.54E-20	G	A	0.15	0.05	0.05	3.57E-01
11	rs11035019	5009563	T	C	0.052	1.010	0.100	5.54E-20	T	C	0.06	0.01	0.08	8.89E-01
11	rs16911934	5251478	C	T	0.057	0.962	0.097	2.19E-19	C	T	0.28	0.07	0.04	7.76E-02
11	rs7929591	5150372	C	T	0.056	0.947	0.096	2.26E-19	C	T	0.23	0.01	0.05	9.11E-01
11	rs16911998	5257700	A	G	0.057	0.962	0.098	2.33E-19	A	G	0.22	0.06	0.05	1.58E-01
11	rs16906901	4901939	G	A	0.052	0.966	0.101	1.42E-18	G	A	0.18	0.09	0.05	7.32E-02
11	rs2133233	5466043	T	C	0.057	0.888	0.097	2.79E-17	T	C	0.07	0.05	0.07	5.23E-01
11	rs7122404	6014364	T	G	0.054	0.891	0.103	6.10E-16	T	G	0.26	-0.06	0.04	2.04E-01
11	rs16924612	4669708	T	C	0.047	0.933	0.107	6.83E-16	T	C	0.24	-0.02	0.04	6.22E-01
11	rs7116019	4618606	G	A	0.047	0.933	0.107	7.06E-16	G	A	0.16	0.09	0.05	9.64E-02

11	rs16913631	6467984	A	C	0.052	0.864	0.106	2.75E-14	A	C	0.11	0.00	0.06	9.93E-01
11	rs6578634	5420909	G	T	0.067	0.731	0.091	4.62E-14	G	T	0.09	-0.05	0.07	4.64E-01
11	rs12226204	6189514	G	A	0.062	0.743	0.096	3.87E-13	G	A	0.17	-0.02	0.05	6.60E-01
11	rs7108599	4607913	A	G	0.094	0.597	0.077	4.18E-13	A	G	0.17	-0.01	0.05	9.04E-01
11	rs11602279	5209592	A	G	0.104	0.562	0.073	4.46E-13	G	A	0.48	-0.03	0.04	3.79E-01
11	rs2167562	3970371	T	C	0.049	0.823	0.108	5.96E-13	T	C	0.17	0.08	0.05	1.06E-01
11	rs16929597	4238136	A	G	0.047	0.844	0.110	6.07E-13	A	G	0.10	0.03	0.06	6.54E-01
11	rs16929572	4238237	C	T	0.047	0.844	0.110	6.07E-13	C	T	0.15	0.03	0.05	6.01E-01
11	rs11033308	4702108	T	C	0.068	0.677	0.091	2.68E-12	T	C	0.10	-0.03	0.06	6.18E-01
11	rs12223580	4706179	A	G	0.068	0.676	0.091	2.87E-12	A	G	0.10	-0.04	0.06	5.75E-01
11	rs4992800	5702462	A	C	0.074	0.640	0.088	5.09E-12	A	C	0.33	0.04	0.04	3.23E-01
11	rs3861787	4647770	T	G	0.095	0.559	0.077	9.04E-12	T	G	0.46	-0.06	0.04	1.53E-01
11	rs3863292	5318677	T	C	0.107	0.525	0.074	1.14E-11	T	C	0.16	-0.05	0.05	2.97E-01
11	rs7950390	4631352	G	T	0.102	0.514	0.075	8.96E-11	G	T	0.47	-0.01	0.04	8.51E-01
11	rs16931173	5469959	C	A	0.120	0.480	0.072	4.07E-10	C	A	0.16	0.04	0.05	4.09E-01

11	rs16907439	4946056	T	C	0.148	0.416	0.065	6.66E-10	T	C	0.09	0.00	0.07	9.66E-01
11	rs7129059	4587940	A	G	0.108	0.467	0.074	1.15E-09	A	G	0.41	-0.02	0.04	6.56E-01
11	rs4269953	5357881	C	T	0.147	0.400	0.064	2.05E-09	T	C	0.41	-0.03	0.04	4.22E-01
11	rs4459298	4596449	A	G	0.153	0.394	0.064	4.15E-09	A	G	0.35	-0.03	0.04	4.40E-01
11	rs1552726	7071031	G	A	0.043	0.748	0.121	4.29E-09	G	A	0.26	0.06	0.04	1.65E-01
11	rs1388526	7020629	A	G	0.057	0.632	0.104	4.55E-09	A	G	0.27	0.02	0.04	5.91E-01
11	rs7951380	4852462	A	G	0.098	0.463	0.076	4.65E-09	A	G	0.17	0.00	0.05	9.31E-01
11	rs16906747	4855124	C	A	0.098	0.463	0.076	4.66E-09	C	A	0.22	0.03	0.04	4.31E-01
11	rs766498	7257474	T	C	0.065	0.575	0.097	8.41E-09	T	C	0.42	0.04	0.04	3.45E-01
11	rs17238860	6242337	C	T	0.091	0.481	0.082	1.07E-08	C	T	0.06	-0.02	0.08	7.84E-01
11	rs2857919	7022160	A	G	0.063	0.578	0.099	1.56E-08	A	G	0.28	0.01	0.04	7.49E-01
11	rs1352860	7020687	C	T	0.063	0.577	0.099	1.66E-08	C	T	0.28	0.02	0.04	6.82E-01
11	rs1124113	4700862	T	C	0.080	0.498	0.086	2.07E-08	T	C	0.12	-0.01	0.06	8.34E-01
11	rs1662162	4115264	T	C	0.147	0.367	0.065	4.45E-08	C	T	0.32	-0.01	0.04	7.95E-01

Supplementary Table 6. Association summary statistics for variants associated with genome-wide significance with mean corpuscular haemoglobin (MCH) in the Pomak cohort and their respective summary statistics in the General Population Cohort (GPC). MCH in the Pomak cohort was inverse-normalised, then z-standardised. MCH in the GPC cohort was inverse-normalised and adjusted for age, age^2 and sex.
EA, effect allele; NEA, Non-effect allele; EAF, Effect allele frequency

**MCH SNPs**			**Pomak**						**General Population Cohort**					
**Chr**	**SNP**	**bp**	**EA**	**NEA**	**EAF**	**BETA**	**SE**	**p value**	**EA**	**NEA**	**EAF**	**BETA**	**SE**	**p value**
11	rs16924612	4669708	T	C	0.046	-0.776	0.111	1.75E-11	T	C	0.240	-0.006	0.045	9.02E-01
11	rs7116019	4618606	G	A	0.046	-0.775	0.111	1.79E-11	G	A	0.156	0.017	0.054	7.54E-01
11	rs12274659	4947444	G	A	0.051	-0.732	0.106	2.38E-11	G	A	0.151	0.028	0.053	6.05E-01
11	rs11035019	5009563	T	C	0.051	-0.732	0.106	2.38E-11	T	C	0.063	0.054	0.084	5.18E-01
11	rs16929597	4238136	A	G	0.046	-0.771	0.113	4.20E-11	A	G	0.101	-0.039	0.064	5.36E-01
11	rs16929572	4238237	C	T	0.046	-0.771	0.113	4.20E-11	C	T	0.151	-0.011	0.054	8.42E-01
11	rs16906901	4901939	G	A	0.051	-0.713	0.106	7.25E-11	G	A	0.180	-0.032	0.051	5.33E-01
11	rs6578634	5420909	G	T	0.066	-0.611	0.093	2.59E-10	G	T	0.091	-0.089	0.067	1.83E-01
11	rs16911934	5251478	C	T	0.056	-0.662	0.102	3.81E-10	C	T	0.280	0.018	0.043	6.69E-01
11	rs16911998	5257700	A	G	0.056	-0.661	0.102	3.91E-10	A	G	0.217	0.034	0.046	4.57E-01

11	rs2167562	3970371	T	C	0.048	-0.697	0.111	9.72E-10	T	C	0.165	0.118	0.051	2.14E-02
11	rs2133233	5466043	T	C	0.056	-0.636	0.101	1.02E-09	T	C	0.071	-0.026	0.074	7.31E-01
11	rs7929591	5150372	C	T	0.055	-0.633	0.101	1.33E-09	C	T	0.227	-0.042	0.045	3.50E-01
11	rs7122404	6014364	T	G	0.053	-0.598	0.107	4.87E-08	T	G	0.258	-0.029	0.044	5.05E-01
11	rs1124113	4700862	T	C	0.078	-0.491	0.088	4.92E-08	T	C	0.121	-0.021	0.059	7.27E-01

Supplementary Table 7. Association at rs7116019 with mean corpuscular volume (MCV), mean corpuscular haemoglobin concentration (MCHC) and mean corpuscular haemoglobin (MCH) in the Pomaks before and after adjustment for the first 10 principal components (PCs).

		Pomak discovery		Pomak replication	
SNP	Trait	P_unadjusted	P_adjusted	P_unadjusted	P_adjusted
rs7116019	MCV	$2.3x10^{-26}$	$1.1x10^{-26}$	$1.6x10^{-14}$	$3.4x10^{-14}$
rs7116019	MCHC	$7.1x10^{-16}$	$2.9x10^{-16}$	$8.6x10^{-15}$	$2.1x10^{-15}$
rs7116019	MCH	$1.8x10^{-11}$	$9.9x10^{-12}$	$1.2x10^{-04}$	$2.1x10^{-04}$

Supplementary Table 8. Enrichment of missense variants among those variants that have increased in frequency above a fold change threshold.

Fold change threshold	N missense > fold change threshold	N missense <= fold change threshold	N other > fold change threshold	N other <= fold change threshold	Enrichment p value	Odds ratio
MANOLIS						
1	4695	4859	313727	321340	6.21E-01	0.99
5	55	9499	1560	633507	3.77E-08	2.35
10	28	9526	489	634578	7.94E-09	3.81
15	16	9538	183	634884	6.48E-08	5.82
20	6	9548	77	634990	1.51E-03	5.18
25	4	9550	33	635034	2.16E-03	8.06
Pomak						
1	4645	4691	303878	322863	1.51E-02	1.05
5	35	9301	878	625863	4.92E-07	2.68
10	7	9329	254	626487	1.13E-01	1.85
15	4	9332	116	626625	1.10E-01	2.32
20	1	9335	65	626676	6.23E-01	1.03
25	1	9335	31	626710	3.77E-01	2.17

Supplementary Table 9. Enrichment of synonymous variants among those variants that have increased in frequency above a fold change threshold in the MANOLIS vs TEENAGE and Pomak vs TEENAGE analyses.

Fold change threshold	N synonymous > fold change threshold	N synonymous <= fold change threshold	N other > fold change threshold	N other <= fold change threshold	p value	Odds ratio
MANOLIS						
1	39414	40794	279008	285405	1.20E-01	0.99
5	249	79959	1366	563047	4.40E-04	1.28
10	89	80119	428	563985	1.67E-03	1.46
15	37	80171	162	564251	1.29E-02	1.61
20	18	80190	65	564348	1.82E-02	1.95
25	9	80199	28	564385	4.17E-02	2.26
Pomak						
1	38133	40843	270390	286711	1.87E-01	0.99
5	128	78848	785	556316	1.45E-01	1.15
10	28	78948	233	556868	4.53E-01	0.85
15	9	78967	111	556990	1.26E-01	0.57
20	3	78973	63	557038	5.93E-02	0.34
25	2	78974	30	557071	4.22E-01	0.47

Supplementary Table 10. Association between genome-wide homozygosity (Fhom), runs of homozygosity (F_ROH) and HELIC study traits at p<0.05 in unrelated individuals from the Pomak population. Respective statistics also shown for available traits in the MANOLIS and TEENAGE populations.
HDL, High density lipoprotein; HOMA-β, Homeostatic model assessment beta cell function; MCH, mean corpuscular haemoglobin;, MCV, mean corpuscular volume.

		Fhom			F_ROH		
**Cohort**	**Trait**	**beta**	**SE**	**p value**	**beta**	**SE**	**p value**
**Pomak**	Height	−19.755	7.306	0.007	-0.092	0.030	0.003
	HDL	−15.588	6.96	0.026	-0.078	0.029	0.007
	HOMA-β	−30.604	15.184	0.046	-0.132	0.064	0.040
	MCH	−14.777	7.051	0.037	-0.057	0.03	0.053
	MCV	−17.875	7.019	0.011	-0.076	0.029	0.011
**MANOLIS**	Height	−4.661	9.138	0.61	-0.018	0.038	0.645
	HDL	−12.905	8.364	0.123	-0.051	0.035	0.139
	HOMA-β	−12.131	10.321	0.24	-0.048	0.042	0.255
	MCH	−7.575	9.232	0.412	-0.036	0.038	0.345
	MCV	−6.768	9.304	0.467	-0.039	0.038	0.304
**TEENAGE**	Height	−11.337	15.597	0.468	0.006	0.147	0.969
	HDL	−14.196	17.163	0.409	0.065	0.164	0.694

Supplementary Table 11. Trait transformation protocol for HELIC-Pomak.

Trait	Abbreviation	Unit	Filter	Gender stratified	Transformation	Covariates	N samples
Body mass index	BMI	kg/m^2	>4xSD	yes	inverse normal	age, age^2	943
C-reactive protein	CRP	mg/L	>3xSD and <0.1 or >10 mg/L	no	inverse normal	-	847
Fasting glucose		mmol/L	>7mmol/L	yes	untransformed	age, age^2	165
Fasting glucose (adjusted for BMI)		mmol/L	>7mmol/L	yes	untransformed	age,age^2, BMI	162
Fasting insulin		μIU/ml	>5xSD	no	inverse normal	-	185
Fasting insulin (adjusted for BMI)		μIU/ml	>5xSD	no	inverse normal	age, age^2, BMI	182
Haemoglobin	Hgb	g/dl	>3xSD	yes	inverse normal	age, age^2	970
Head circumference		cm	>4xSD	yes	untransformed	age	856
Height		cm	>4xSD	yes	inverse normal	age, age^2	944
High-density lipoprotein	HDL	mmol/L	>5xSD	yes	inverse normal	-	987
Hip circumference		cm	>4xSD	yes	inverse normal	age, age^2	894
Hip circumference (adjusted for BMI)		cm	>4xSD	yes	inverse normal	age, age^2, BMI	882
Homeostatic model assessment insulin resistance	HOMA-IR		>5xSD	no	inverse normal	-	183
Homeostatic model assessment insulin resistance (adjusted for BMI)	HOMA-IR		>5xSD	no	inverse normal	age, age^2, BMI	180
Homeostatic model assessment β cell function	HOMA-β		>5xSD	yes	inverse normal	age, age^2	182
Homeostatic model assessment β cell function (adjusted for BMI)	HOMA-β		>5xSD	yes	inverse normal	age, age^2, BMI	179
Low-density lipoprotein	LDL	mmol/L	>5xSD	yes	inverse normal	age, age^2	987
Mean corpuscular haemoglobin	MCH	pg	>3xSD	yes	inverse normal	-	960
Mean corpuscular haemoglobin concentration	MCHC	g/dl	>3xSD	yes	untransformed	age, age^2	974
Mean corpuscular volume	MCV	fl	>3xSD	yes	inverse normal	-	965
Packed cell volume	PCV	%	>3xSD	yes	inverse normal	-	973
Platelets	PLT	10^9/L	>3xSD	yes	inverse normal	-	979
Red blood cells	RBC	10^12/L	>3xSD	yes	inverse normal	-	979
Sitting height		cm	>4xSD	yes	untransformed	age	929

Total cholesterol	TC	mmol/L	>5xSD	no	inverse normal	age, age^2	970
Triglycerides	TG	mmol/L	>5xSD	yes	log	age, age^2, fasting	975
Waist circumference		cm	>4xSD	yes	inverse normal	age, age^2	898
Waist circumference (adjusted for BMI)		cm	>4xSD	yes	inverse normal	BMI	886
Waist hip ratio	WHR	w(cm)/h(cm)	>4xSD	yes	inverse normal	age, age^2	890
Waist hip ratio (adjusted for BMI)	WHR	w(cm)/h(cm)	>4xSD	yes	inverse normal	age, age^2, BMI	878
Weight		kg	>4xSD	yes	inverse normal	age, age^2	953
White Blood Cells	WBC	10^9/L	>3xSD	yes	log	-	974

Supplementary Table 12. Trait transformation protocol for HELIC-MANOLIS.

Trait	Abbreviation	Unit	Filter	Gender stratified	Transformation	Covariates	N samples
Birth weight		kg	>4xSD	no	inverse normal	age, age^2	49
Body mass index	BMI	kg/m^2	>4xSD	no	inverse normal	age, age^2	1033
C-reactive protein	CRP	mg/L	>3xSD and <0.1 or >10 mg/L	no	inverse normal	age, age^2	1108
Diastolic blood pressure	DBP	mmHg	>5xSD	no	inverse normal	BMI	580
Fasting glucose		mmol/L	>7mmol/L	yes	inverse normal	age, age^2	727
Fasting glucose (adjusted for BMI)		mmol/L	>7mmol/L	yes	inverse normal	age, age^2, BMI	641
Fasting insulin		µIU/ml	>5xSD	no	inverse normal	age, age^2	827
Fasting insulin (adjusted for BMI)		µIU/ml	>5xSD	no	inverse normal	BMI	731
Gestation age		months	>4xSD	no	inverse normal	age, age^2	341
Haemoglobin	Hgb	g/dl	>3xSD	yes	inverse normal	age, age^2	1008
Head circumference		cm	>4xSD	yes	untransformed	age	1069
Height		cm	>4xSD	yes	inverse normal	age, age^2	1058
High-density lipoprotein	HDL	mmol/L	>5xSD	yes	inverse normal	-	1264
Hip circumference		cm	>4xSD	yes	inverse normal	age, age^2	1065
Hip circumference (adjusted for BMI)		cm	>4xSD	yes	inverse normal	age, age^2, BMI	1013
Homeostatic model assessment insulin resistance	HOMA-IR		>5xSD	no	inverse normal	age, age^2	826
Homeostatic model assessment insulin resistance (adjusted for BMI)	HOMA-IR		>5xSD	no	inverse normal	age, age^2, BMI	732
Homeostatic model assessment β cell function	HOMA-β		>5xSD	no	inverse normal	age, age^2	832
Homeostatic model assessment β cell function (adjusted for BMI)	HOMA-β		>5xSD	no	inverse normal	age, age^2, BMI	735
Low-density lipoprotein	LDL	mmol/L	>5xSD	yes	inverse normal	age, age2	1270
Mean corpuscular haemoglobin	MCH	pg	>3xSD	yes	inverse normal	age, age^2	995
Mean corpuscular haemoglobin concentration	MCHC	g/dl	>3xSD	yes	untransformed	-	1008

Mean corpuscular volume	MCV	fl	>3xSD	yes	inverse normal	age, age^2	993
Packed cell volume	PCV	%	>3xSD	yes	inverse normal	age, age^2	1007
Platelets	PLT	10^9/L	>3xSD	yes	inverse normal	age, age^2	1005
Red blood cells	RBC	10^12/L	>3xSD	yes	inverse normal	-	1003
Sitting height		cm	>4xSD	yes	inverse normal	age, age^2	942
Systolic blood pressure	SBP	mmHg	>5xSD	no	inverse normal	age, age2, BMI, gender	580
Total cholesterol	TC	mmol/L	>5xSD	no	inverse normal	age, age2	1255
Triglycerides	TG	mmol/L	>5xSD	yes	log	age, age2, fasting	1262
Waist circumference		cm	>4xSD	yes	inverse normal	age, age^2	1075
Waist circumference (adjusted for BMI)		cm	>4xSD	yes	inverse normal	age, age^2, BMI	1020
Waist hip ratio	WHR	w(cm)/h(cm)	>4xSD	yes	inverse normal	age, age^2	1068
Waist hip ratio (adjusted for BMI)	WHR	w(cm)/h(cm)	>4xSD	yes	inverse normal	age, age^2, BMI	1016
Weight		kg	>4xSD	yes	inverse normal	age, age^2	1066
White Blood Cells	WBC	10^9/L	>3xSD	yes	log	-	1004

Supplementary Table 13. The table shows the emission scores for the hidden Markov model (HMM). Let x (in {0,1,2}) be the genotype of the query individual at a site and m and f the genotypes of the pseudo-mother and pseudo-father respectively. Then x, m and f are compatible if x could have plausibly been inherited from m and f. Otherwise, the emission score is penalised by ε (~0.01) if only one of the parents is incompatible or ε^2 if both are incompatible with x.

	x = 0			x=1			x=2		
f/m	0	1	2	0	1	2	0	1	2
0	1	1	ε	ε	1	1	ε^2	ε	ε
1	1	1	ε	1	1	1	ε	1	1
2	ε	ε	ε^2	1	1	ε	ε	1	1

Supplementary Note 1. Isolate age

We used two different methods to estimate the age of the isolates (Supplementary Methods). Using the method described in McEvoy *et al*. (2011)[1] we estimated divergence time of 39 generations (~1000 years with generation time of 25 years) for MANOLIS and TEENAGE and 52 generations (~1300 years; generation time 25 years) for Pomak and TEENAGE. This translate in both MANOLIS and Pomak separating very recently from TEENAGE, however these estimates might have been lowered by migration, which is not directly taken into account in this approach.

To estimate the age of separation between the isolated and TEENAGE populations we also used the extension to the Long-Range Phasing (LRP) method[2] (Supplementary Methods) which considers recent common ancestors between Pomak and TEENAGE and between MANOLIS and TEENAGE. The median age is estimated to be 109 and 106 generations respectively, suggesting that an upper bound for the time of isolation is 19 generations and 16 generations respectively. Note that these estimates assume a simple model in which separation is a single event, the effective population size (Ne) of the TEENAGE population has remained constant over time and that there has been no gene flow between the two isolates and the general Greek population since isolation began. For example, the data may also be compatible with a model of older divergence with a low level of more recent immigration.

Supplementary Note 2. Genetic drift

Allele frequency spectra at the intersection of variants between MANOLIS and TEENAGE and between Pomak and TEENAGE show that the three Greek populations have similar allele frequency distributions for common variants (Supplementary Fig. 8). We observe a lower proportion of monomorphics in MANOLIS than in TEENAGE (0.29% vs 1.11%) while the opposite is true for rare [minor allele frequency (MAF)<0.01] (3.46% vs 2.65% respectively) and for low frequency [MAF=1-5%] variants (7.32% vs 6.62% respectively) (Supplementary Fig. 8A). Similarly we observe a lower proportion of monomorphics in Pomak than in TEENAGE (0.39% vs 0.69%) but a larger proportion of rare (2.56% vs 1.82% respectively) and low frequency variants (7.22% vs 6.53%) (Supplementary Fig. 8B).

We observe that a larger number of monomorphic and rare variants in TEENAGE have increased frequency in each of the isolates: 15,341 (2.35% of the total number of variants examined) have increased in frequency in MANOLIS with respect to TEENAGE against 8,197 (1.26%) which have decreased in frequency; 8,908 (1.39% of the total number of variants examined) have increased in frequency in the Pomak with respect to TEENAGE against 6,154 (0.96%) of variants which have decreased in frequency) (Supplementary Table 3). For variants that are monomorphic or rare in TEENAGE we observe mean absolute allele frequency increases of 0.006 and 0.007 in MANOLIS and Pomak respectively.

In contrast to the absolute allele frequency analyses fewer variants show large fold differences in Pomak vs TEENAGE than MANOLIS vs TEENAGE. This could be due to sample size differences between the cohorts; large fold allele frequency increases in the isolates are observed mostly at variants that are rare in the outbred population but the 20% decrease in the sample size of the Pomak vs TEENAGE cohorts could be responsible for rare variants in

TEENAGE being unobserved in the Pomaks; the sample size of MANOLIS is comparable to TEENAGE (6% more samples in MANOLIS than in TEENAGE).

Supplementary Note 3. Power calculations

MANOLIS. For the variant that has risen in frequency by 0.000038 we have 35% power to detect an effect size of 1 in MANOLIS compared to 34.64% power in the outbred Greek population. For the variant that has risen in frequency by 0.01 in MANOLIS we have 78.97% power to detect this in MANOLIS as opposed to 2.95% in the outbred Greek population. For the variant that increased by 0.06897 we have 99.99% power to detect an effect size of 1 as opposed to 0% power in the outbred Greek population. Therefore the power gains to detect a rare variant that has risen up in frequency in our isolated population compared to an outbred population range from 0.36-99.99%. We repeated these calculations by fixing the sample size to that of the unrelated individuals from MANOLIS (N=754) and we find that for the variant that has risen in frequency by 0.000038 we have 5.87% power to detect an effect size of 1 in MANOLIS compared to 5.78% power in the outbred Greek population. For the variant that has risen in frequency by 0.01 in MANOLIS we have 25.69% power to detect this in MANOLIS unrelated as opposed to 0.33% in the outbred Greek population. For the variant that increased by 0.06897 we have 99.99% power to detect an effect size of 1 as opposed to 0% power in the outbred Greek population. This results in power gains ranging from 0.1%-99.99%.

Pomak. 16,105 of the variants that overlap between TEENAGE and Pomak were rare in TEENAGE. Of these 8,906 (55.3%) have risen in frequency in the Pomak and 3,342 (18%) have reached MAF>0.01. Allele frequency increases range from 0.000608 (MAF in Pomak=0.01058; MAF in TEENAGE=0.009972) to 0.0885 (MAF in Pomak=0.0885; MAF in TEENAGE=0) with a median increase of 0.01237 (MAF in Pomak=0.01675, MAF in TEENAGE=0.004958). We calculated power as above (except we fixed the sample size to the size of the Pomak population N=1,014) at allele frequencies that corresponded to the minimum, median and maximum values of this range. For the variant that has risen in frequency by 0.000608 we have 20.63% power to detect an effect size of 1 in the Pomaks compared to 16.99% power in the outbred Greek population. For the variant that has risen in frequency by 0.01237 in the Pomak we have 64.67% power to detect this in MANOLIS as opposed to 1.13% in the outbred Greek population. For the variant that increased by 0.0885 we have 99% power to detect an effect size of 1 opposed to 0% power in the outbred Greek population. Therefore the power gains to detect a rare variant that has risen up in frequency in the Pomak population compared to the outbred Greek population range from 3.64-99.99%. We repeated these calculations by fixing the sample size to that of the unrelated individuals from Pomak (N=567) and we find that for the variant that has risen in frequency by 0.000608 we have 2.34% power to detect an effect size of 1 in the Pomak unrelated individuals compared to 1.84% power in the outbred Greek population. For the variant that has risen in frequency by 0.01237 in the Pomak we have 13.72% power to detect this in Pomak unrelated individuals as opposed to 0.1% in the outbred Greek population. For the variant that increased by 0.0885 we have 99.99% power to detect an effect size of 1 opposed to 0% power in the outbred Greek population. This results in power gains ranging from 0.5%-99.99%.

Supplementary Note 4. Haplotype structure

The shared haplotype in all Pomak chromosomes with the minor allele of rs7116019, rs12274659 and rs11035019 is about 1.8Mb in size. The two homozygous individuals for the minor allele of the more distant SNP, rs16913631, are not shared with the three individuals who are homozygous for the other three SNPs. One of the Luhya (LWK) haplotypes carrying rs7116019G shared about 183kb, but the two TEENAGE chromosomes only share about 7kb. For rs12274659G and rs11035019T, one LWK haplotype shared about 230kb, and for TEENAGE no data were available (Supplementary Fig. 10). The high frequency and diversity of haplotypes carrying these derived alleles in the LWK suggest that they arose in Africa and entered Europe later. In Europe, the Pomak haplotype has a very different structure and likely different origin from the TEENAGE haplotype. We therefore propose a model where haplotypes carrying these alleles have entered European populations more than once. Most relevant here is that a different haplotype entered the Pomak population compared with the general Greek population.

Supplementary Methods

Extension of the Long-Range Phasing approach

We used a method, which is an extension of Kong *et al.*'s (2008)[2] LRP approach, to identify, for each individual at each location in the genome, the two other individuals across the data set, to whom they are most closely related; their genealogical nearest neighbours (NNs). To identify NNs for a query individual we construct a hidden Markov model (HMM) where the hidden states are all the pairs of individuals in the sample, acting as candidate pseudo-parents similar to the LRP. The observed states are comprised of the genotype of the query individual. Transitions model recombination events. If r is the probability of recombination between two sites (assumed here constant for simplicity) and n is the number of haplotypes, then for distinct individuals a, b, c, d the probability of transitioning from pair i to j, $t_{i,j}$ is as follows:

$$t_{i,j} = \begin{cases} (1-r)^2 + O\left(1/n\right), & i = \{a,b\}, j = \{a,b\} \\ \dfrac{r(1-r)}{n} + O\left(r^2/n^2\right), & i = \{a,b\}, j = \{a,d\} \\ \dfrac{r^2}{n(n-1)} \quad , & i = \{a,b\}, j = \{c,d\} \end{cases} \tag{1}$$

Emissions are the compatibility between the query genotype and the candidate parents, shown in Supplementary Table 13. The HMM structure allows to use the Viterbi algorithm to obtain a maximum likelihood estimate for the sequence of NNs across the genome. In practice, however, in order to achieve computational efficiency we use a series of heuristics to constrain the algorithm.

We reconstruct shared haplotype lengths by observing the genomic stretches over which NNs do not change. By examining the physical and genetic lengths of haplotype sharing within and between populations, we can, moreover, estimate the average date at which these common ancestors lived (TMRCA). Our estimate of the TMRCA between NNs from the same sample, T say, can be used to obtain a moment estimator for the effective population size, Ne, of the underlying population. If n is the number of haplotypes in the sample, then the TMRCA between NNs, t say, is 2/n. Then the number of generations to the TMRCA is t*2Ne and therefore a moment-estimator for Ne is T/(2t) = 4T/n.

TMRCA analysis

Haplotype sharing between two individuals around a particular locus is expected to decay exponentially, with parameter twice the number of generations to their TMRCA times the genetic distance away from the locus. By examining how far away from a particular locus the NNs change on average, we obtain an estimate for the decay around that locus. Using the half-life of this decay we infer the TMRCA at that position and by sampling different positions across the genome we obtain an estimate for the genome-wide average TMRCA. We employ this approach to date the TMRCA between MANOLIS samples (8.65 gens), between POMAK samples (8.56 gens) and between the TEENAGE individuals (89.7 gens). By examining haplotype sharing between the isolate individuals and the TEENAGE samples, we may similarly date the co-ancestry between them. The median age is estimated to be 109 and 106 generations between MANOLIS and TEENAGE and between Pomak and TEENAGE respectively. These estimates allow us to heuristically date the time when isolation began: an upper bound for this time is 19 generations and 16 generations for the MANOLIS and

Pomak cohorts respectively. An illustration of the demographic scenario assumed here, as well as the calculations involved can be found in Supplementary Fig. 12. Note that these estimates assume a simple model in which separation is a single event, the effective population size of the TEENAGE population has remained constant over time and that there has been no gene flow between the two isolates and the general Greek population since isolation began. For example, the data may also be compatible with a model of older divergence with a low level of more recent immigration.

Isolate age

Divergence time between pairs of populations was obtained as described in McEvoy *et al.*[1] using Fst information and the harmonic mean of Ne estimates of the last 800 generations. In isolated populations, founder effects and small population size can have a dramatic effect on the level of genetic variation. While measures of allelic differentiation, such as Fst, identify such effects, they are largely uninformative about the age of co-ancestry between individuals in a population. Isolate age was also estimated with the extension to the LRP approach as described in the preceding sections.

Power calculations

Power was calculated using Quanto v1.2.4[3] assuming a population mean of 0 and a standard deviation (SD) of 1. We calculated power (at the genome-wide significance threshold $5x10^{-8}$) to detect an effect size of 1SD (which for a variant of MAF 0.01 would explain 2% of the trait variance) by fixing the sample size to the size of the MANOLIS population N=1,282 and of the Pomak population N=1,014. We also calculated power by fixing the sample size to that

of unrelated individuals from the MANOLIS and Pomak cohorts (N=754 and N=567 respectively).

Trait transformations (MANOLIS, Pomak and TEENAGE)

Our phenotype preparation protocol involves filtering out values that were at least 3 standard deviations away from the mean, and then phenotype normalisation (where required) within gender in the cases that gender was statistically significant (Mann-Whitney test $p<0.05$) (Supplementary Tables 11 and 12 for Pomak and MANOLIS respectively). Using the normalised phenotype, we performed in R a simple linear regression to adjust for age and age-squared within gender, in the cases where age was statistically significant. The regression residuals were z-standardized within gender and then combined across gender. z-standardisation transforms the residuals so that they have a mean of 0 and a standard deviation of 1; this allows them to be comparable across gender.

Replication datasets

Pomak replication dataset, genotyping and quality control

DNA samples from the Pomak replication collection were genotyped using Illumina HumanCoreExome-12v1-0_A (Illumina, San Diego, USA) at the Wellcome Trust Sanger Institute, Hinxton, UK. Genotypes were called using GenCall (Illumina Genome Studio) followed by zCall[4] and quality control (QC) was performed in two stages (pre- and post-zCall). In the pre-zCall QC, the initial dataset comprised 824 Pomak individuals and 538,448 variants. After performing an initial removal of samples and variants with call rate <90%, samples underwent standard QC procedures, with exclusion criteria as follows: i) sample call

rate <98%; ii) samples with sex discrepancies; iii) samples who were visual outliers for autosomal heterozygosity (calculated separately for variants with MAF<1% and MAF≥1%); iv) duplicate samples identified by calculating the pairwise identity by descent (IBD) for each sample using PLINK v1.07[5]; from each pair with a pi-hat>0.9 the sample with the lower call rate was excluded; v) samples with evidence of non-European descent or outliers from the main cluster as assessed by multidimensional scaling (MDS) analysis in PLINK[5] by combining each population with populations from 1000 Genomes[6]; vi) Sequenom concordance. 73 samples that didn't pass the criteria were excluded and to improve rare variant calling the missing genotypes were called using zCall. Post-zCall variant exclusion criteria were as follows. GenCall variant based: i) call rate <95% ii) Hardy Weinberg Equilibrium (HWE) exact p<0.0001. zCall sample based: i) sample call rate <99%; ii) Autosomal heterozygosity (separately for variants with MAF<1% and MAF≥1%) visual outliers excluded; iii) Visual outliers from the distribution of the number of singleton variants for each sample excluded. ZCall variant based: i) call rate <99% ii) HWE exact p<0.0001 iii) cluster separation score <0.4. The resulting dataset comprised 740 individuals and 529,086 variants.

The General Population Cohort Study

The General Population Cohort Study **(**GPC)[7] is a population-based open cohort of approximately 22,000 people living within 25 neighbouring villages of the Kyamulibwa sub-county of Kalungu district in rural south-west Uganda. The cohort was established in 1989 by Medical Research Council (MRC) UK in collaboration with the Uganda Virus Research Institute (UVRI) to examine trends in prevalence and incidence of HIV infection and their determinants.

The GPC population is assessed through annual house-to-house 'rounds' of census and survey, during which demographic, medical and serological data are collected. The GPC Round 22 used for GWAS analysis contained five main stages which took place in 2011 over the course of the year; mobilisation (recruitment and consenting), mapping, census, survey, and feedback of results and clinical follow-up. This study was approved by the Science and Ethics Committee of the UVRI, the Ugandan National Council for Science and Technology, and the East of England-Cambridge South (formerly Cambridgeshire 4) (National Health Service) NHS Research Ethics Committee UK.

GPC genotyping, quality control and association analyses

Ugandan participants were genotyped on the HumanOmni2.5-8 Illumina genotyping chip (Illumina, San Diego, USA) at the Wellcome Trust Sanger Institute, Hinxton UK. Genotypes were called using the Illuminus genotype calling algorithm[8]. Samples underwent standard QC procedures, with exclusion criteria as follows: i) sample call rate <97%; ii) samples with sex discrepancies; iii) samples who were outliers for autosomal heterozygosity (mean±3SD); iv) duplicated samples (pi-hat>0.90) identified by calculating the pairwise IBD for each sample using PLINK[5]; v) ethnic outliers as assessed by principal component analysis (PCA) using EIGENSOFT[9,10] by combining the GPC cohort with populations from 1000 Genomes[6]. SNP exclusion criteria were as follows: i) call rate <97% ii) Hardy Weinberg Equilibrium (HWE) exact $p<10^{-8}$. A total of 4,778 individuals and 2,340,487 SNPs passed QC in the GPC cohort. 1,479 individuals and 1,844,709 autosomal SNPs were tested for association with mean corpuscular volume (MCV), mean corpuscular haemoglobin (MCH) and mean corpuscular haemoglobin concentration (MCHC) using an exact mixed-model approach to account for both subtle relatedness and population stratification implemented in GEMMA

v0.93[11]. MCV, MHC and MHCH traits were inverse normalised and regressed on age, age^2 and sex.

Population stratification

We performed MDS analysis in PLINK[5] as described in Methods separately for the Pomak discovery (N=98,517 SNPs) and Pomak replication (N=81,651 SNPs) datasets and generated the first 10 principal components. To account for population stratification we repeated the association analysis at rs7116019 with MCV, MHC, and MCHC using GEMMA[11] by including the first 10 principal components as covariates.

Haplotype structure analysis

The shared haplotype block carrying rs7116019G, rs12274659G and rs11035019T among the Pomak individuals was first defined using three unrelated individuals who were homozygous for this haplotype, and then refined by examining the haplotype length shared with other unrelated individuals who were heterozygous for these three SNPs. Using the same strategy, we also identified the longest block shared between Pomak and LWK in 1000 Genomes and TEENAGE haplotypes.

Simulations

We used simuPOP[12] to simulate data for a population with the Pomak demographic parameters estimated from this study (Supplementary Fig. 11). We simulated an initial allele frequency of 0.01 at one locus immediately after the split from the parental population,

which we then follow for 52 generations; in addition we assumed a mutation rate of $2x10^{-8}$ per nucleotide per generation. We simulated 100,000 replicates in each scenario.

Supplementary References

1 McEvoy, B. P., Powell, J. E., Goddard, M. E. & Visscher, P. M. Human population dispersal "Out of Africa" estimated from linkage disequilibrium and allele frequencies of SNPs. *Genome Res.* **21**, 821-829, doi:10.1101/gr.119636.110 (2011).

2 Kong, A. *et al.* Detection of sharing by descent, long-range phasing and haplotype imputation. *Nat. Genet.* **40**, 1068-1075, doi:10.1038/ng.216 (2008).

3 Gauderman, W. & Morrison, J. QUANTO 1.1: A computer program for power and sample size calculations for genetic-epidemiology studies http://hydra.usc.edu/gxe. (2006).

4 Goldstein, J. I. *et al.* zCall: a rare variant caller for array-based genotyping: genetics and population analysis. *Bioinformatics (Oxford, England)* **28**, 2543-2545, doi:10.1093/bioinformatics/bts479 (2012).

5 Purcell, S. *et al.* PLINK: a tool set for whole-genome association and population-based linkage analyses. *Am. J. Hum. Genet.* **81**, 559-575, doi:10.1086/519795 (2007).

6 1000 Genomes Project Consortium *et al.* An integrated map of genetic variation from 1,092 human genomes. *Nature* **491**, 56-65, doi:10.1038/nature11632 (2012).

7 Asiki, G. *et al.* The general population cohort in rural south-western Uganda: a platform for communicable and non-communicable disease studies. *Int J Epidemiol* **42**, 129-141, doi:10.1093/ije/dys234 (2013).

8 Teo, Y. Y. *et al.* A genotype calling algorithm for the Illumina BeadArray platform. *Bioinformatics* **23**, 2741-2746, doi:10.1093/bioinformatics/btm443 (2007).

9 Patterson, N., Price, A. L. & Reich, D. Population structure and eigenanalysis. *PLoS Genet.* **2**, e190, doi:10.1371/journal.pgen.0020190 (2006).

10 Price, A. L. *et al.* Principal components analysis corrects for stratification in genome-wide association studies. *Nat. Genet.* **38**, 904-909, doi:10.1038/ng1847 (2006).

11 Zhou, X. & Stephens, M. Genome-wide efficient mixed-model analysis for association studies. *Nat. Genet.* **44**, 821-824, doi:10.1038/ng.2310 (2012).

12 Peng, B. & Kimmel, M. simuPOP: a forward-time population genetics simulation environment. *Bioinformatics (Oxford, England)* **21**, 3686-3687, doi:10.1093/bioinformatics/bti584 (2005).
